# Knowledge-based in silico fragmentation and annotation of mass spectra for natural products with MassKG

**DOI:** 10.1016/j.csbj.2024.09.001

**Published:** 2024-09-07

**Authors:** Bingjie Zhu, Zhenhao Li, Zehua Jin, Yi Zhong, Tianhang Lv, Zhiwei Ge, Haoran Li, Tianhao Wang, Yugang Lin, Huihui Liu, Tianyi Ma, Shufang Wang, Jie Liao, Xiaohui Fan

**Affiliations:** a College of Pharmaceutical Sciences, Zhejiang University, Hangzhou 310058, China; b State Key Laboratory of Chinese Medicine Modernization, Innovation Center of Yangtze River Delta, Zhejiang University, Jiaxing 314100, China; c Analysis Center of Agrobiology and Environmental Sciences, Zhejiang University, Hangzhou 310058, China; d Zhang Boli Intelligent Health Innovation Lab, Hangzhou 311121, China; e Department of Pharmacy, Affiliated Jinhua Hospital, Zhejiang University School of Medicine, Jinhua 321000, China; f The Joint-laboratory of Clinical Multi-Omics Research between Zhejiang University and Ningbo Municipal Hospital of TCM, Ningbo Municipal Hospital of TCM, 315100 Ningbo, China

**Keywords:** Mass spectrometry, Natural products, In silico fragmentation, MS annotation, Deep learning

## Abstract

Liquid chromatography coupled with tandem mass spectrometry (LC-MS/MS) is a potent analytical technique utilized for identifying natural products from complex sources. However, due to the structural diversity, annotating LC-MS/MS data of natural products efficiently remains challenging, hindering the discovery process of novel active structures. Here, we introduce MassKG, an algorithm that combines a knowledge-based fragmentation strategy and a deep learning-based molecule generation model to aid in rapid dereplication and the discovery of novel NP structures. Specifically, MassKG has compiled 407,720 known NP structures and, based on this, generated 266,353 new structures using chemical language models for the discovery of potential novel compounds. Furthermore, MassKG demonstrates exceptional performance in spectra annotation compared to state-of-the-art algorithms. To enhance usability, MassKG has been implemented as a web server for annotating tandem mass spectral data (MS/MS, MS2) with a user-friendly interface, automatic reporting, and fragment tree visualization. Lastly, the interpretive capability of MassKG is comprehensively validated through composition analysis and MS annotation of *Panax notoginseng, Ginkgo biloba*, *Codonopsis pilosula*, and *Astragalus membranaceus*. MassKG is now accessible at https://xomics.com.cn/masskg.

## Graphical Abstract

**Figure:**
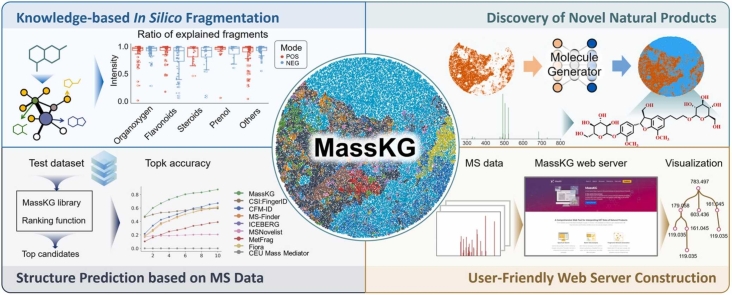

## 1 Introduction

Natural products (NPs) have historically proven their value as a source of molecules with therapeutic potential, and nowadays still represent an important pool for the identification of novel drug leads [1], [2], [3]. Among the state-of-the-art analytical techniques, liquid chromatography coupled to tandem mass spectrometry (LC-MS/MS) using electrospray ionization (ESI) method is widely employed for the characterization of the NPs with diverse structures [4], [5]. However, annotating the complex MS/MS (MS^2^) spectra generated from naturally occurring mixtures remains a challenge. Traditional manual annotation involves inferring molecular formulas, retrieving candidates from databases, and ranking them based on MS^2^ spectra similarity. This process requires specialized knowledge and is time-consuming, limiting the efficiency of structure elucidation. The ideal annotation method is to compare the experimental MS/MS spectra to the reference spectral libraries such as HMDB [6], METLIN [7], GNPS [8], [9], and MassBank [10]. However, the inevitable limitation of database-searching based strategy is the poor availability of MS^2^ spectra database [11]. In addition, the MS^2^ spectra databases of ESI source are hardly standardized consequence of the influence from instrument type, acquisition condition and sample variations [12]. Consequently, identifying compounds from accurate MS^2^ data remains a bottleneck in untargeted metabolomics [13], [14], [15].

To address this challenge, focus has shifted towards computational tools for efficient large-scale sample annotation. The in silico fragmentation strategy summarized the observed fragmentation rules to predict the fragments for small molecule identification from MS^2^ data. Met-Frag [16] is one of the first in silico fragmentation approaches which applied a top-down approach, starting with an entire molecular graph and removing each bond successively [17]. MS-Finder [18] is also a rule-based in silico fragmentation method that applied nine hydrogen (H) rearrangement rules to simulate the possible fragments and their mass-to-charge ratio (*m/z*). Magma [17] is another tool that yields a hierarchical tree of substructures of a candidate molecule to explain the fragment peaks observed at consecutive levels of the multistage MS(n) spectral tree. Beside these, with the advance of artificial intelligence, machine-learning (ML) based algorithms gradually emerged. One of the famous tool is CSI-FingerID [19] (has been integrated into SIRIUS [20] workflow), which use fragmentation trees to predict molecular fingerprints by multiple kernel learning, and further ranking the retrieved candidates from structure databases as PubChem [21] and Chemspider [22] by fingerprint comparison. Besides predicting fragments, there are algorithms try to predict the in-silico spectra. CFM-ID [23] is a ML model to simulate the in silico spectra of small molecules. The original version of CFM-ID applied a generative Markov model, while the newest version employed a graph model and assisted with hand-written-rules. Spectra prediction also attracted the attention of complicated transformer-structured models such as Massformer [24], and ICEBERG [25], but these two models only perform spectra prediction in positive ion mode.

Beyond annotation known molecules, efforts have also made to identify novel structures from MS^2^ data. CANOPUS [26] used a deep neural network to directly predict chemical classes for unknown structures. The FSEA algorithm, which serves as an enrichment analysis tool integrated into MS-DIAL, is also able to recommend metabolite classes for unknowns [27]. In addition, database of hypothetical compound structures generated by modifying known metabolite structures offered opportunities for discovering unknown structures [28], [29]. COSMIC [30] applied such strategy and annotated 12 natural bile acids that were never reported before based on the generated bile acid conjugates. Deep learning models with an encoder-decoder structure can also be used to generate novel structures. The representative study using this strategy is MSNovelist, which directly infer novel chemical structures from the MS^2^ data of compounds based on the structural fingerprints predicted by SIRIUS and CSI: FingerID and structure generation through an encoder-decoder model [31]. Skinnider et al. raised a data augmentation strategy to train a robust generation model (called chemical language model in that paper) using a relatively small dataset [32]. Then, coupling the generation models with in silico MS^2^ spectra prediction enables automated structure elucidation of novel psychoactive substances [33]. In the field of NP research, the application of LC-MS/MS requires rapid dereplication of known structures and provides insight into potential novel structures [34]. While discovering new structural compounds with activity has always been the core goal of natural product research [35], it is essential to explore methods for novel structure investigation [36].

In this work, we propose MassKG, an algorithm that combines a knowledge-based fragmentation strategy and a deep learning-based molecule generation model to assist in automatic MS2 data annotation of complex NP samples. The term "KG" specifically refers to "knowledge" and should not be confused with its common interpretation as "knowledge graph." MassKG extracts fragmentation knowledge through statistical analysis of reported fragmentation patterns from an open-source curated MS^2^ library of NPs. The largest open-source NP database, COCONUT [37], containing over 400,000 structures, serves as the candidate source for MassKG and is also used to train RNN-based molecule generation models to create NPs with novel structures. Both known, and newly generated structures are subsequently subjected to a fragmentation process to develop the MS^2^ fragment database. This enables MassKG to rapidly dereplicate known NPs and assist in the discovery of novel NPs by annotating MS^2^ data from complex sources. For convenience, MassKG is implemented as an online web platform with a user-friendly interface, allowing users to easily execute an automatic annotation process, with results visualized in the form of a fragmentation graph and an annotation report.

## 2 Material and methods

### 2.1 Dataset preparation

The training dataset for MS^2^ spectra library of NPs were collected from GNPS [8], encompassing 8782 spectra of natural products in positive ion mode from 5302 unique compounds and 2351 spectra from 1820 unique compounds (Supplementary File 1–2). Datasets for test and validation were manually curated in-house datasets. Experimental method for sample preparation and LC-MS/MS condition is listed in Supplementary material 1.1 and 1.2. The test dataset (Supplementary File 3–4) is comprised of 314 MS^2^ spectra in positive mode and 320 spectra in negative mode of 257 NPs, compound information is listed in Supplementary File 5. The validation and application dataset of herbal extracts are also collected experimentally with same LC-MS/MS condition with the test dataset.

Furthermore, metadata of NPs with structures reported by SMILES code was gathered from the COCONUT database, which contains over 400,000 unique NPs from 52 different sources, including compounds from a wide variety of organisms (mainly plants), geographic locations and applications [37]. NPs in COCONUT database contain chemical ontology assigned by ClassyFire [38], covering Kingdom, Superclass, Class, and Subclass. In addition, COCONUT database provides annotation levels for each natural product with 1–5 stars.

### 2.2 Extraction fragmentation knowledge by statistical analysis

Given a specific collision energy, collision-induced dissociation (CID) breaks the precursor ion into smaller fragments, including both charged and neutral pieces. Therefore, the MS^2^ spectra of a molecule are determined by three factors: 1) which bond(s) are cleaved, 2) how H rearrangement occurs, and 3) which fragment retains the charge after bond cleavage. To elucidate the specific rules of NP fragmentation, including bond cleavage patterns, H transition patterns, and charge assignment patterns, we conducted a statistical analysis on the GNPS MS^2^ library and synthesized the statistical results with expert knowledge to build the knowledge-based fragment generator.

1) MassKG can break up to two non-cyclic bonds or four cyclic bonds simultaneously. In its current version, MassKG considers only molecules composed of the elements C, H, O, N, P, and S, resulting in a theoretical total of 15 classes of atom pair combinations to represent a bond.
2) H rearrangement patterns were statistical analyzed of the 15 bonds. For a bond cleavage, the minimum of H rearrange number was set 0 and maximum was determined by the bond type, where single bond is 1, double bond and aromatic bond is 2. Then the statistical result was summarized as an experimental formula to calculate H rearrangements.
3) Charge position was also summarized by statistical analysis, considering the correlation between atom composition and fragment charge or not.

### 2.3 Generating in silico fragment library by knowledge-based model

The extracted fragmentation knowledge was used to construct a in silico fragment generator was employed to create an in silico fragment library of NPs. The structural information of NPs used in the current version of MassKG is sourced from the COCONUT database. SMILES of the NPs were used as the input of the trained fragment generator to obtain the predicted fragment sets for each molecule. Subsequently, we calculated the exact molecular weights of these fragments and simulated their MS^2^ spectra based on H rearrangement and electron distribution principles. This process allowed us to construct a comprehensive fragment library for all NPs recorded in COCONUT, which can be utilized for annotating the experimental MS^2^ spectra.

### 2.4 Inference of molecular formulas

A reference molecular formula library that includes the molecular formulas and corresponding exact masses for the molecules recorded in COCONUT. The candidate formulas were calculated according to the error between the experimental precursor mass-to-charge ratio (*m/z*) and the reference based on a user-defined mass error tolerance. The mass error score was calculated as:

$$\mathrm{Mass error score}=e^{-0.5*{\left(\frac{{\mathrm{mass}}_{\exp}-{\mathrm{mass}}_{\mathrm{theor}}}{\delta }\right)}^{2}}$$

where ${\mathrm{mass}}_{\exp}$ is the experimental precursor *m/z* and ${\mathrm{mass}}_{\mathrm{theor}}$ is the calculated theory *m/z* according to formula. δ is the user defined mass error tolerance, with a default setting of 5 mDa.

In the current version, the formula library composed of elements of C, H, O, N, P, and S. The ion type of [M + H]^+^, [M-H_2_O + H]^+^, [M + Na]^+^, [M + K]^+^, [M + NH_4_]^+^ are supported for positive ion mode, and [M-H]^-^, [M + FA-H]^-^, [2M-H]^-^, [M + Cl]^-^ for negative ion mode.

Further, we collected a set of frequent neutral losses (Supplementary File 6) and a neutral loss score was calculated as below:

$$\mathrm{neutral loss score}=\frac{1}{2}\left(\sum S_{\mathrm{intensity}}S_{\mathrm{MES}}+\sum T_{\mathrm{intensity}}T_{\mathrm{MES}}\right)$$

where $S_{\mathrm{intensity}}$ and $S_{\mathrm{MES}}$ denote the normalized intensity and the mass error score of the source peaks and $T_{\mathrm{intensity}}$ and $T_{\mathrm{MES}}$ denote those of the target peaks.

Note that formula inference is not the core task of MassKG. Instead, it serves as a complementary tool for queries when the molecular formula is unavailable.

### 2.5 Retrieving and ranking candidates

Based on the inferred formulas, the corresponding structures were retrieved from the candidate database, which encompassed both metadata and structural information. Subsequently, candidates were ranked by several score functions. Initially, the intensity of the matched peaks was determined:

$$S_{1}=\sum {\mathrm{Peak}}_{\mathrm{intensity}}$$

where ${\mathrm{Peak}}_{\mathrm{intensity}}$ represented the normalized peak intensity of matched fragments, the maximum of $S_{1}$ is 1. Then the total mass error score (MES) of fragments was considered:

$$S_{2}=\frac{\sum {\mathrm{MES}}_{i}}{N}$$

where i represents the index of matched peaks and N is the total number of matched peaks. The maximum value of $S_{2}$ is 1. Next, the cosine similarity between predicted and reference spectra is considered. To achieve this, we trained a random forest model using the MACCS keys of the molecule, along with the bond features as well as the related atom features as inputs using the pervious LC-MS/MS dataset of our lab (Supplementary File 7). The inverse of the relative peak intensity was used as the output to simulate a continuous value of bond dissociation energy (BDE). Details regarding the feature embeddings are provided in the Supplementary material 4.1. The cosine similarity is calculated using the CosineGreedy function of matchms toolkits [39]. The maximum of cosine similarity ($S_{3}$) is also 1.

In addition to scores related to matched fragments, the metadata of candidates is also taken into account. We designed a metascore ($S_{4}$) correlation to the annotation level of candidates in COCONUT database, which defined as $\frac{\mathrm{level}}{5}$. Since the level is a discrete value ranging from 0 to 5, $S_{4}$ adopts the values in the set {0, 0.2, 0.4, 0.6, 0.8, 1}. Further, for LC-MS/MS data, a retention time (RT) score can also be considered. The RT of unknown metabolites can be predicted by a trained random forest model using MACCS key as inputs. Then a RT score can be calculated as:

$$S_{5}=\frac{1}{1+e^{\left(\frac{{\mathrm{RT}}_{\exp}-{\mathrm{RT}}_{\mathrm{pred}}}{\theta -1}\right)}}$$

where ${\mathrm{RT}}_{\exp}$ and ${\mathrm{RT}}_{\mathrm{pred}}$ represent the experiment and predicted RTs. θ is the set maximum error in minute between ${\mathrm{RT}}_{\exp}$ and ${\mathrm{RT}}_{\mathrm{pred}}$，here θ is set to 0.1. Please note that the current RT prediction model is trained on our LC condition using the same data as the BDE model. If the user intends to add a RT score but has a different LC condition, a linear correction is recommended.

To sum up, the final score was defined as the sum of the above-mentioned scores:

$$F\mathrm{inal score}=S_{0}+\alpha S_{1}+\beta S_{2}+\gamma S_{3}+S_{4\;}{+\;S}_{5}$$

where$\;S_{0}$ denotes the formula score, which equal to the sum of neutral loss score and MES of precursor *m/z*. $S_{1}$ denotes the structure score, $S_{2}$ denotes MES of fragments, $S_{3}$ denotes the cosine similarity, $S_{4}$ denotes the meta score and $S_{4}$ denotes the RT score. α,βand γ coefficients between 0 and 1 and were optimized by a grid search method (see Supplementary material 4.2). Finally, α was set to 0.1, β was set to 0.2 and γ was set to 0.1. All retrieved candidates were then ranked according to the final score.

### 2.6 Baseline tools

MetFrag, CFM-ID, and MAGMA were used as the baseline tools to evaluate the fragmentation performance of MassKG. In addition, despite of MetFrag and CFM-ID, MSFINDER, CSI:FingerID (integrated into SIRIUS), ICEBERG, MSNovelist (integrated into SIRIUS), Fiora [40], CEU Mass Mediator [41] and feature-based molecular network (FBMN) [9] were applied as the baselines to evaluate the metabolites annotation performance of MassKG. In details, MetFrag implemented by the MetFragR package (version 2.2); CFM-ID (version 4.0) was implemented by a compiled program [42]; results from MSFINDER (version 3.52) and SIRIUS (version 6.0.2) were directly acquired by the running the software [18], [20]. Trained models of Fiora and ICEBERG were run locally to predict the spectra of candidates, and candidates were ranked by a cosine similarity between experimental and the predicted spectra. Results of CEU Mass Mediator and FBMN were observed by their web service.

### 2.7 RNN-based molecule generator

RNN-based chemical language models [32], [33] were employed to construct the molecule generator for yielding the chemical space of generated new NP structures. Canonical SMILES of the known NPs were used as the inputs. In this study, we utilized both a three-layer LSTM model and a GRU model. The architecture of these models consisted of a hidden layer with 512 dimensions, an embedding layer with 128 dimensions, and no dropout layers. The models were trained using the Adam optimizer with parameters β1 = 0.9 and β2 = 0.999, a batch size of 128, and a learning rate of 0.001. The entire set of 407,270 SMILES representations of known NPs was used to train these models. Subsequently, the chemical space of the generated NPs was compared with that of the known NPs.

### 2.8 Metrics for systematical evaluation

The performance of fragment generation is evaluated based on the proportion of explained peak intensities, which is consistent with the calculation of the structure score. The accuracy of annotations is assessed using top accuracy. Receiver operating characteristic curve (ROC) of annotation accuracy is plotted by setting thresholds on structural similarity. The efficiency of MassKG is evaluated by the average time consumption for candidates. The Evaluation metrics for the molecular generator were based on the methods described by Skinnider et al. [32] Additional metrics for evaluating the molecule generation model were listed in Supplementary material.

### 2.9 Web server implementation

The metabolite annotation pipeline was implemented via a user-friendly web server. MassKG was constructed using the Flask framework (version 2.2.2). The back-end infrastructure of MassKG relies on MySQL for data storage and retrieval. The front-end of the application was developed using HTML, CSS, and JavaScript. The algorithm of MassKG was developed in Python 3.7.9, including, knowledge-based fragmentation, deep-learning-based models for molecule generation, candidate rank score calculation, fragment tree construction and so on.

## 3 Results

### 3.1 Design concept of MassKG

Though NP structures are in high diversity, there are common fragmentation reactions for ions generated by ESI in positive and negative modes using collision cells, making it possible to build a knowledge-based fragmentation and annotation tool to assist MS^2^ data analysis [43]. The goal of MassKG is to use programming langrage represent the know knowledges, building a robust model to generate in silico fragments of both known and undiscovered NPs.

**Fig. 1:**
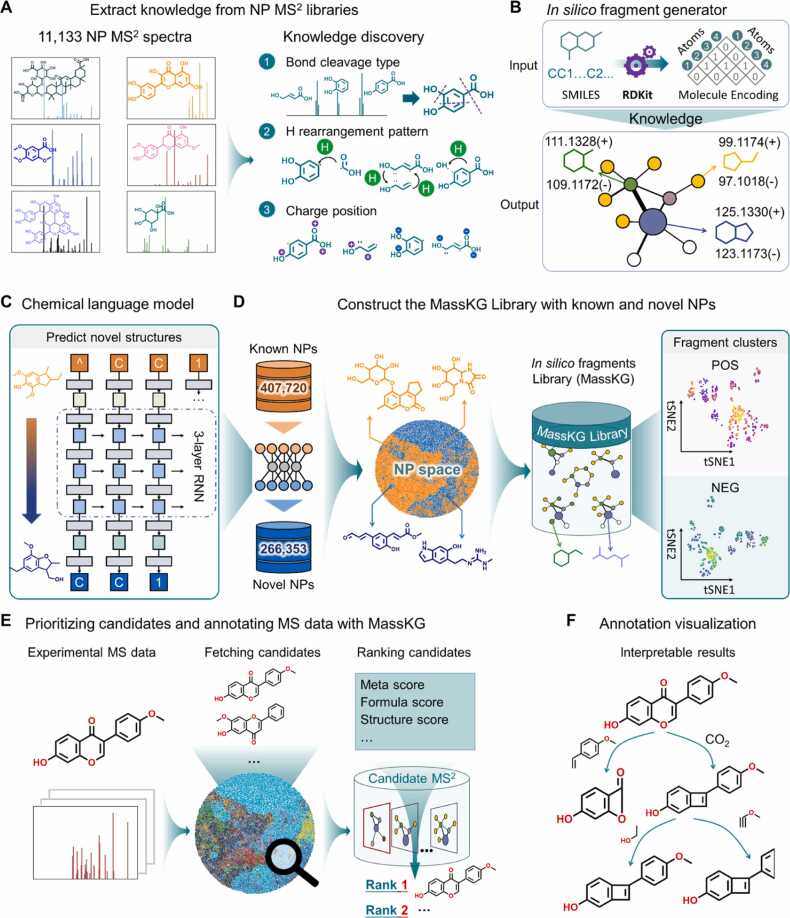
**Scheme of MassKG.** (A) Schematic illustration of the process of training the fragment generator from open source MS^2^ libraries. (B) Workflow of the knowledge-based fragment generator which predict the in silico fragments of given structures based on the extracted knowledge. (C) The structure of RNN-based chemical language model to predict novel NP structures. (D) Construction of in silico fragment library, which consists of the known and generated NP structures. (E) The schematic illustration of the annotation of experimental MS^2^ data using MassKG. (F) The fragment tree of annotation results for visualization.

The workflow of MassKG is illustrated in Fig. 1, and it can be divided into four primary components: extracting fragmentation knowledge from open-source MS2 libraries (Figs. 1A and 1B), utilizing chemical language models as a molecule generator to predict potential novel NP structures (Fig. 1C), establishing the MassKG in silico MS^2^ library, which includes both known and novel NP structures generated by the trained fragment generator (Fig. 1D), and prioritizing candidates and annotating experimental MS^2^ data with MassKG (Fig. 1E). The annotation results are visualized as a fragment tree (Fig. 1F).

The first step involves statistical analysis of known fragmentation patterns to determine the types of bond cleavage, rules of H rearrangement, and charge assignment based on pattern frequency. The second step involves gathering a large dataset of known natural product structures to train a molecule generation model, simulating new NP structures, and providing insights for discovering new structural compounds. The third step uses a knowledge-based fragmentation model to predict in silico fragmentation of both known and generated compounds, constructing a large-scale natural product fragment library for MS^2^ data annotation. The final step retrieves candidates for given experimental MS^1^ and MS^2^ data, ranking them based on a comprehensive score that includes molecular formula score, structure annotation score, meta score, etc. The ultimate result is presented as a fragment cleavage tree, where fragments are connected by neutral losses, providing an explanation of the fragment relationship for each compound. Since MassKG's candidates consist of both known and undiscovered molecules, it has the potential for rapid dereplication of known NPs and aiding in the discovery of new compounds.

### 3.2 Building the in-house dataset using ESI-TOF MS/MS

**Fig. 2:**
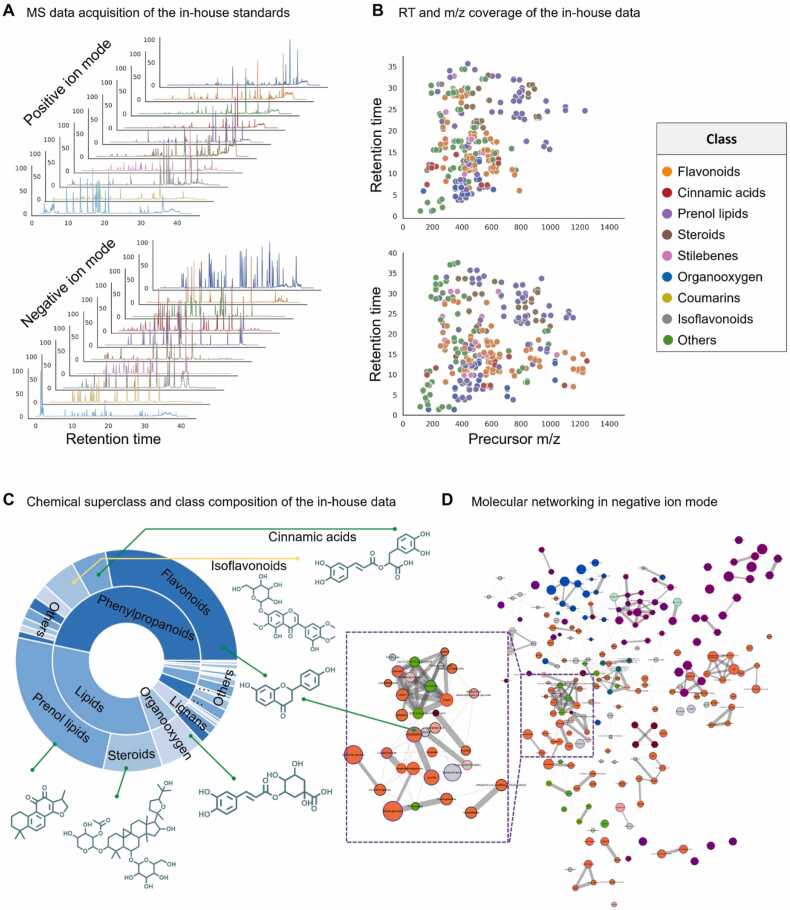
**Overview of the in-house dataset.** (A) The base peak chromatogram (BPC) of the in-house MS^2^ data. (B) Distribution of *m/z* and RT of the standard compounds in positive ion mode (top) and negative ion mode (bottom) respectively. (C) The chemical ontology of the in-house dataset. (D) The distribution of NP clusters within a molecular network derived from the negative mode of MS^2^ spectra (refer to Fig. S1 for the positive mode), with a detailed enlargement of a subnetwork primarily consisting of *Flavonoids* as a representative example.

The distribution of these compounds across RT and *m/z* was relatively uniform, indicating that the data is representative and suitable for subsequent rule validation and model evaluation studies (Fig. 2B). Notably, compounds with RT within 20 min were more prevalent than those with RT between 20 and 40 min. This observation suggests that the in-house dataset contains a higher proportion of high-polarity compounds compared to low-polarity compounds. The *m/z* distribution was concentrated between 150 and 1300 *m/z*, with a higher abundance of compounds below 600 *m/z* compared to those above 600 *m/z*. This indicates that the dataset primarily consists of small molecule metabolites. Additionally, the observed ion types of the precursors includes 137 [M + H]^+^, 107 [M + Na]^+^, 39 [M+NH_4_]^+^, and 31 [M-H_2_O]^+^ in the positive ion mode while 177 [M-H]^-^, 91 [M + FA-H]^-^, and 52 [2M-H]^-^ in the negative ion mode.

Raw MS^2^ data was annotated and curated manually to yield the in-house MS^2^ spectra dataset, which comprises 314 MS^2^ spectra acquired in positive ion mode and 320 spectra in negative ion mode. The number of spectra exceeds that of standards due to the possibility of multiple ion types generated by the same compound. The standards primarily encompass three superclasses: *Phenylpropanoids and polyketides*, *Lipids*, and *Organic oxygen compounds*. The *Phenylpropanoids and polyketides* superclass is further categorized into *Flavonoids*, *Isoflavonoids*, and *Cinnamic acids*. The *Lipids* superclass consists of *Prenol lipids* and *Steroids*. The *Organic oxygen compounds* superclass solely comprises the *Organooxygen compounds* class. More specifically, the positive ion mode dataset contains 152 *Phenylpropanoids and polyketides*, 103 *Lipids and lipid*, 23 *Organic oxygen compounds* for the superclass, and further breakdowns of 87 *Flavonoids*, 75 *Prenol lipids*, 28 *Steroids and steroid derivatives*, 23 *Organooxygen compounds*, 19 *Cinnamic acids and derivatives*, 12 *Isoflavonoids*, and 8 *Coumarins and derivatives*. Similarly, the negative ion mode dataset includes 144 *Phenylpropanoids and polyketides*, 110 *Lipids and lipid*, 19 *Organic oxygen compounds* for the superclass, and further breakdowns of 91 *Flavonoids*, 83 *Prenol lipids*, 27 *Steroids and steroid derivatives*, 19 *Organooxygen compounds*, 13 *Cinnamic acids and derivatives*, 20 *Isoflavonoids*, and 6 *Coumarins and derivatives*. Total statistic result of chemical ontology is depicted in Fig. 2C. The in-house dataset will serve as the test dataset for evaluating the performance of the trained fragment generator and the annotation accuracy of MassKG.

### 3.3 Knowledge extraction from training dataset

**Fig. 3:**
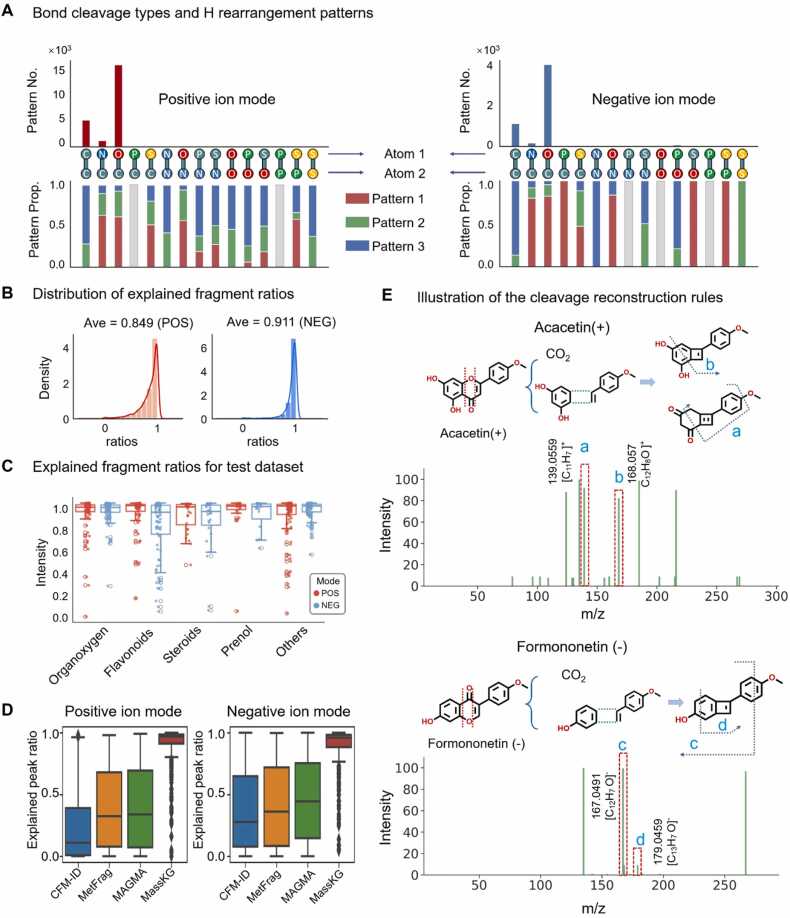
**Description of the knowledge-driven fragment generator**. (A) The statistical results for the total bond cleavage types (top) and the proportions of different of H rearrangement patterns (bottom) for both positive and negative ion modes. (B) Distribution of the explained MS^2^ fragments ratio by MassKG in training dataset. The average of explained fragment ratio in positive mode (red) is 0.849 and negative mode (blue) is 0.911. (C) Distribution of the explained fragment ratio of test dataset with different chemical classes in positive mode (red) and negative mode (blue). (D) Comparison of peak annotation performance of MassKG, CFM-ID, MetFrag and MAGMA. (E) Examples to explain the supplementary cleavage-reconstruction rules specific to Flavonoids. The red dashed lines indicate bond cleavage, and green dashed lines represent the reconstruction process. Fragments observed only by the supplementary rules are circled with red dashed lines. Their specific fragmentation patterns are traced with blue dashed lines, and the corresponding MS^2^ peaks are labeled with blue letters. AVE: average. Prop.: proportion.

We counted the frequency of different bond cleavage types of the GNPS dataset and found that C-O, C-C, and C-N bond cleavages were the most prevalent in both ion modes, accounting for 97.7 % in positive and 97.9 % in negative ion mode. Notably, C-O bond cleavage stood out as the dominant type, comprising 69.8 % in positive and 74.2 % in negative ion mode (top panels of Fig. 3A).

Furthermore, we delved into the H rearrangement patterns observed in these bond cleavages. We identified three patterns to describe the H rearrangement: Pattern 1 represents H atoms shifting from the atom1-terminal fragment to the atom2-terminal fragment, Pattern 3 represents the opposite, and Pattern 2 represents homolytic cleavage without H rearrangement. Our results revealed specific trends in H rearrangement. In the positive ion mode, of the 16,470 C—O bond cleavages, Pattern 1 was observed in 10,126 cases, indicating an H shift from the C-terminal to the O-terminal fragment. Pattern 2 occurred in 5131 cases, and Pattern 3 in 1213 cases. For C—C bonds, Pattern 1 was identified in 3851 cases, with Pattern 2 in 1509. In the case of C—N bonds, Pattern 1 was found in 779 out of 1232 cases, Pattern 2 in 328, and Pattern 3 in 125 (Fig. 3A, bottom left). In the negative ion mode, Pattern 1 was present in 3190 of the 3886 C—O bond cleavages, Pattern 2 in 521, and Pattern 3 in 175 cases. Among the 1085 C—C bond cases, Pattern 1 was observed in 941, and Pattern 2 in 144. For the 157 C—N bond cases, there were 125 instances of Pattern 1, 19 of Pattern 2, and 13 of Pattern 3 (Fig. 3 A, bottom right).

Based on these observations, we propose a rule for H rearrangement in MassKG fragment generation, grounded in atomic electronegativity. Specifically, H atoms tend to shift from fragment terminals with higher electronegativity to those with lower electronegativity. This rule held true in 93.7 % of positive ion mode cases and 96.2 % of negative ion mode cases, indicating its robustness and utility in predicting and understanding NP fragmentation patterns.

Subsequently, we formulated a streamlined H rearrangement principle employing a "rank value"(RV) that correlates with element electronegativity. This ranking value serves as a proxy for the likelihood of H rearrangement during bond cleavage. Based on this principle, we devised a function that accurately describes H rearrangement, thereby enhancing our understanding of NP fragmentation patterns. Let X and Y represent atom1 and atom2 respectively, the possible H rearrangement pattern were calculated as fellow:

$$\left\{\begin{array}{c} \begin{array}{c} H_{x}=n*\left\{-1,0,+1\right\}\;R\left(X\right)=R\left(Y\right)\\ H_{x}=n*\left\{0,+1\right\}\;R\left(X\right)>R\left(Y\right) \end{array}\\ H_{x}=n*\left\{-1,0\right\}\;R\left(X\right)<R\left(Y\right)\\ H_{y}=n-H_{x}\; \end{array}\right)$$

here, R(X) and R(Y) represents the RV of X and Y, which were dependent on the atom electronegativity. Specifically, in this study, O and N were assigned a RV of 2 and 1, respectively, while other atoms including C, P and S were assigned RV of 0. This means that R (O) = 2, R (N) = 1, R (C) = R(P) = R(S) = 0. The variable $H_{x}$ represents the number of H atoms to be transferred from Y to X. A value of − 1 indicates that one H atom would be shifted from X to Y (Pattern 1), + 1 indicates the opposite direction (Pattern 3), and 0 means no H rearrangement, resulting in two radical fragments (Pattern 2). n represents the type of bond and its value is 1 for single bond and 2 for double bond.

We subsequently analyzed the proportions of carbon-only fragments in both ion modes, finding that they accounted for 23.6 % in the positive mode and only 1.5 % in the negative mode. Consequently, we have restricted MassKG from generating fragments containing heteroatoms in the negative mode during fragmentation, while no such restrictions apply in the positive mode.

### 3.4 Performance evaluation of MassKG

#### 3.4.1 Spectra interpretation ability of MassKG

The GNPS dataset contains 519, 348 fragments in positive mode and 46, 424 fragments in negative mode, of which 84. 9 % (positive ion mode) and 91.1 % (negative ion mode) were explained by MassKG (Fig. 3B). Further MassKG was tested using our in-house dataset, which was composed of 5637 fragments of 314 spectra for positive and 5616 fragments of 320 spectra for negative. As a result, in positive and negative mode, 90.1 % and 88.8 % of the fragments were accurately resolved. Then the results of NPs in different chemical classes were evaluated separately, where the average ratio of explained fragments for *Flavonoids*, *Prenol lipids*, *Steroids and steroid derivatives Organooxygen compounds* were 90.9 %, 91.6 %, 88.6 %and 91.8 % in positive ion mode and 92.9 %, 79.5 %, 82.3 %, 89.9 % in negative ion mode respectively (Fig. 3C). Details of the relationship between molecule *m/z* and generated fragments were described in Supplementary material 3. The peak annotation performance was further compared with three well-known in silico fragmentation tools: MetFrag, CFM-ID, and MAGMA. The results indicate that MassKG achieved the highest peak interpretation ratio (Fig. 3D). However, when viewed as a whole, some fragments still exhibited poor interpretability (the outliers). Then we consider a supplementary rule named cleavage-reconstruction rule, which allowed a molecule cleavage into fragments and then reform a substructure with losing a small neutral piece, was established to explain the unmatched peaks. For example, the reconstruction of a four-membered ring after the loss of CO_2_ from the C-ring would be observed in the *Flavonoids* and this reaction was called a “1,3 elimination reaction”. The pattern of reconstruction successfully matched the peaks with *m/z* 139.0559 and *m/z* 168.057 of Acacetin (Fig. 3. E, top) in positive ion mode and *m/z* 167.0491 and *m/z* 179.0459 of Formononetin in negative ion mode (Fig. 3. E, bottom).

#### 3.4.2 Annotation accuracy of MassKG

**Fig. 4:**
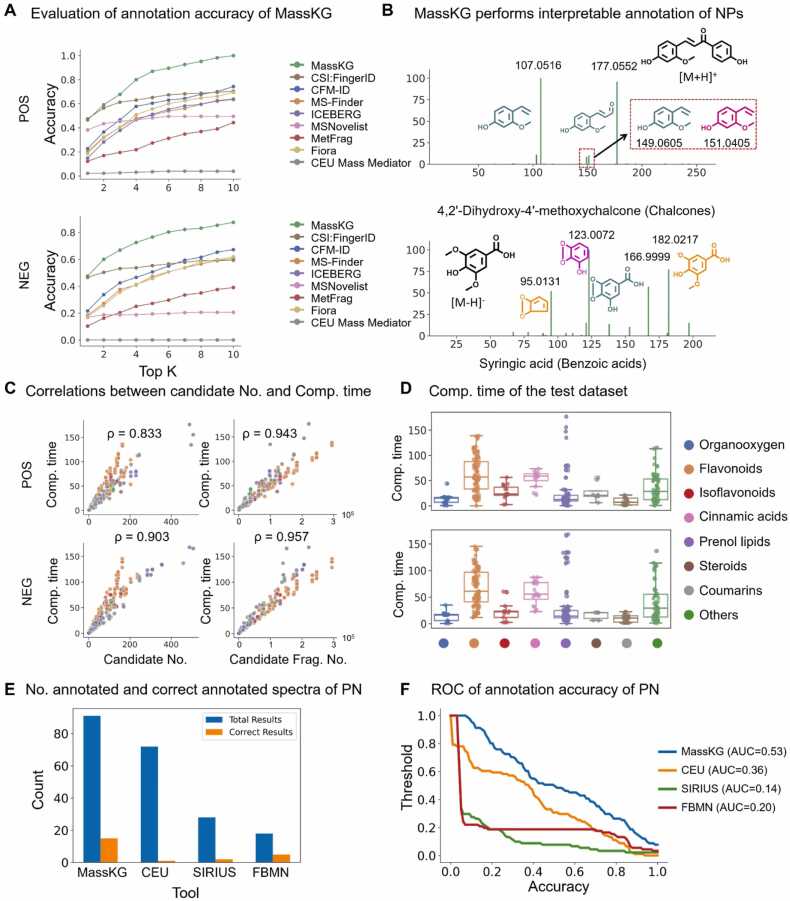
**Performance evaluation of the MassKG.** (A) Top-k accuracy of MassKG compared with the baseline methods. (B) Examples of interpretation MassKG annotation results. The prototype structures are colored in black, fragments conform to Pattern 1 are colored in light green, fragments conform to Pattern 3 are colored in pink and fragment conform to Pattern 2 are colored in yellow. (C) Estimation of the computational time of MassKG, showcase the correlations between computation time, number of candidates and number of candidate fragments. (D) Computational time distribution for each class in test dataset. ρ: Pearson correlation coefficient. (E) The number of total annotated and correct annotated spectra of the PN dataset. (F) ROC curve of annotation accuracy of PN dataset with different threshold of structural similarity.

The annotation accuracy of MassKG were compared with various baseline tools including MetFrag, CFM-ID, MSFINDER, CSI:FingerID, ICEBERG, MSNovelist, Fiora and CEU Mass Mediator. The MS1 tolerance was set 5 mDa or 10 ppm and the MS2 tolerance was set 20 mDa or 20 ppm. In both positive and negative ion mode, MassKG gained the best performance (Fig. 4A). In positive ion mode, the top 1, top 3 and top 10 accuracy of MassKG was 46.5 %, 67.8 %, and 98.2 % respectively. In negative ion mode, the top 1, top 3 and top 10 accuracy was 47.5 %, top 3 was 66.9 %, and top 10 was 87.5 % respectively. Among the baseline tools, CSI:FingerID exhibited the highest accuracy, with a top 1 accuracy of 47.4 % in positive mode, slightly surpassing MassKG. However, as the k value increased, MassKG demonstrated superior performance. The CEU Mass Mediator exhibited the lowest accuracy, ranging from 0.02 to 0.04 for top 1 to top 10 predictions. Additionally, since ICEBERG was unable to predict spectra in negative mode, its accuracy was recorded as 0. In addition, with continuous BDE, MassKG now is able to predict spectra to some extent, part of the well predicted spectra is shown in Fig. S4–S5.

Importantly, since MassKG is a knowledge-based model, it provides interpretable annotation results. To illustrate this, we present two annotation cases using MassKG. The first case involves 4,2′-Dihydroxy-4′-methoxychalcone, which belongs to the class of Chalcones. In positive ion mode, MassKG successfully explained its main fragments. It can be observed that this compound exhibits the typical phenomenon of different mass spectrum peaks from the same fragment. Both *m/z* 149.0605 and *m/z* 151.0405 originate from the same fragment, resulting from the cleavage of a C—C bond, and the specific *m/z* is determined by H rearrangement. In negative ion mode, we consider Syringic acid, which belongs to Benzoic acids. MassKG annotated its four main fragment peaks. Among them, *m/z* 182.0217 is obtained by the loss of CH_3_∙ from the parent ion, while *m/z* 166.9999 represents a fragment generated by the loss of two methyl groups from the parent ion. *m/z* 123.0072 is produced by the subsequent loss of a CO_2_ molecule, and further loss of a CO molecule results in the fragment *m/z* 95.0131 (Fig. 4B).

#### 3.4.3 Time consumption

Furthermore, we tested the annotation performance of MassKG. First, we evaluated the running speed of MassKG on a Linux server equipped with 256 GB of memory and 96 cores. The spectrum matching speed correlates with the number of candidates for the compound, with correlation coefficients of 0.833 and 0.903 in positive and negative ion modes, respectively (Fig. 4C, left). For the total fragments of candidates, time consumption had correlation coefficients of 0.943 in positive mode and 0.957 in negative mode (Fig. 4C, right). The average computation time for each candidate is approximately 0.3 s.

#### 3.4.4 Systematic validation of MassKG using herb dataset

A MS^2^ dataset of *Panax notoginseng* (PN) was acquired under the same LC-MS/MS conditions and manually annotated the compounds within it. This dataset serves as an independent dataset for systematic validation of MassKG (Supplementary File 8). The systematic validation directly annotates the MS^2^ data without confirmed formula information. Top 1 annotation accuracy is applied as the metrics. The PN dataset includes a total of 91 NP structures. As a result, MassKG successfully annotated all 91 queries, while CEU Mass Mediator annotated 72, Sirius annotated 28, and FBMN annotated 18 queries. The number of correct annotated compounds for each tool was 15 for MassKG, 1 for CEU, 2 for Sirius, and 5 for FBMN, with MassKG showing the best performance (Fig. 4E). Furthermore, we plotted the ROC curve based on the annotation results, as shown in Fig. 4F. The results indicate that MassKG also performed the best, with an AUC of 0.54, while the other tools had AUCs of 0.36 for CEU, 0.14 for SIRIUS, and 0.20 for FBMN.

### 3.5 Generate fragments for novel structures using MassKG

**Fig. 5:**
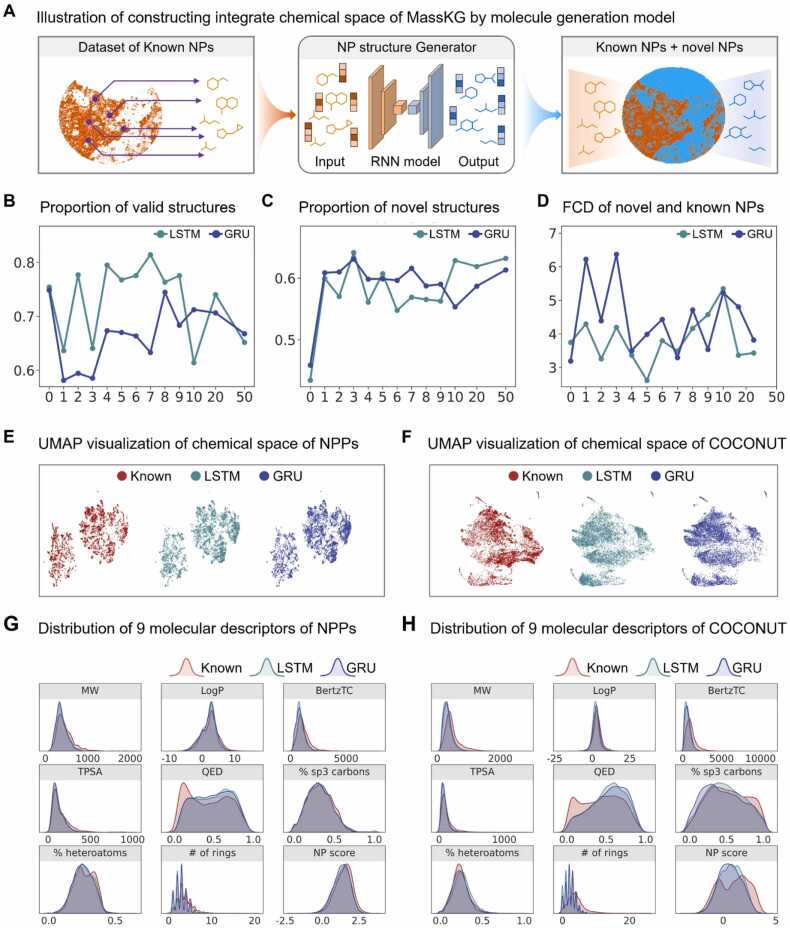
**Performance evaluation of the MassKG in generating novel structures from known NPs.** (A) Schematic illustration of the RNN model-based molecule generator. Panels B, C, and D exhibit metrics for RNN-based models trained on 18,297 NPPSs, which are the proportion of valid SMILES strings, the proportion of novel SMILES strings, and the FCD to the training set, respectively, across varying degrees of non-canonical SMILES generation. Panel E offers a UMAP visualization comparing known NPPSs with an equal number of randomly sampled molecules generated by trained LSTM and GRU models (left: known NPSs, middle: LSTM-generated molecules, right: GRU-generated molecules). Similarly, panel F provides a UMAP visualization for NPs from the COCONUT database versus randomly sampled generated molecules from the trained LSTM and GRU models (same order as panel E). Panels G and H detail descriptors for known NPPSs and COCONUT NPs, respectively, alongside the generated molecules. These descriptors include molecular weight, LogP, TPSA, the proportion of sp3-hybridized carbons, the proportion of heteroatoms, the number of rings, QED, BertzTC, and the natural product-likeness score.

The molecule generation model is structured in RNN, with known molecules and inputs and novel molecules as outputs (Fig. 5A). When using synthetic compound structures as the training set, the performance of the molecular generation model is affected by the size of the dataset^31^. To test whether this effect still exists when using NPs as the training set, we first selected a smaller dataset (composed of 18,297 NPs mainly synthesized via the phenylpropanoid-shikimate pathway (NPPSs) (Fig. 5B–D). We compared the performance of molecule generation models with data augmentation multiples ranging from 1 to 50, evaluating them based on three metrics: the percentage of valid structures (% Valid), the percentage of novel structures (% novel), and Fréchet ChemNet Distance (FCD). The results showed that varying the data augmentation degree had little impact on the performance of either the LSTM or GRU model. This may be because compounds generated through the same metabolic pathway tend to have similar structures, limiting the model's ability to explore a wider chemical space even with a larger dataset. To explore a wider range of molecules, we trained another LSTM and GRU model using all 407,720 known molecular structures as input. Data augmentation was not applied in this step due to the already large dataset size.

As a result, more than 500,000 molecules were generated. Among these, approximately 53.7 % were identified as valid molecules. Compared to the training dataset, 92.5 % of the molecules were newly generated, resulting in 266,353 unique new molecules after deduplication. The statistical results including KL divergence, Jensen-Shannon distances and Wasserstein distance between the whole generated molecules and real molecules can be found in the Supplementary Table S1.

Furthermore, a random sample of 10,000 molecules was compared to the feature distributions of molecules in the training dataset. We used CDDD model [44] to embed SMILES into 512-dimensional vectors and then reduced to 2-dimensional vectors using UMAP for visualization (Fig. 5E and Fig. 5F). The distributions of nine molecular descriptors, including molecular weight (MW), LogP, topological polar surface area (TPSA), percentage of sp3-hybridized carbons (% sp3 carbons), percentage of heteroatoms (% heteroatoms), number of rings (# of rings), Quantitative Estimate of Drug-likeness (QED), BertzCT, and natural product-likeness score (NP score), were compared between the generated molecules and the known molecules in the training set to statistically assess their similarity. As shown in Fig. 5G and Fig. 5H, the generated NP molecules shared similar structural features with those in the training dataset. This observation is consistent with the general trend in NP research, where specific types of compounds share common characteristics, such as similar molecular skeletons and functional groups. Then, the generated molecules with characteristics similar to those of known NPs were collected, and in silico MS^2^ spectra were predicted using the aforementioned fragment generator of MassKG. The in silico MS^2^ of generated NPs together with the known NPs constructed the integrate MS^2^ library of MassKG.

### 3.6 Applying MassKG to discover novel compounds

**Fig. 6:**
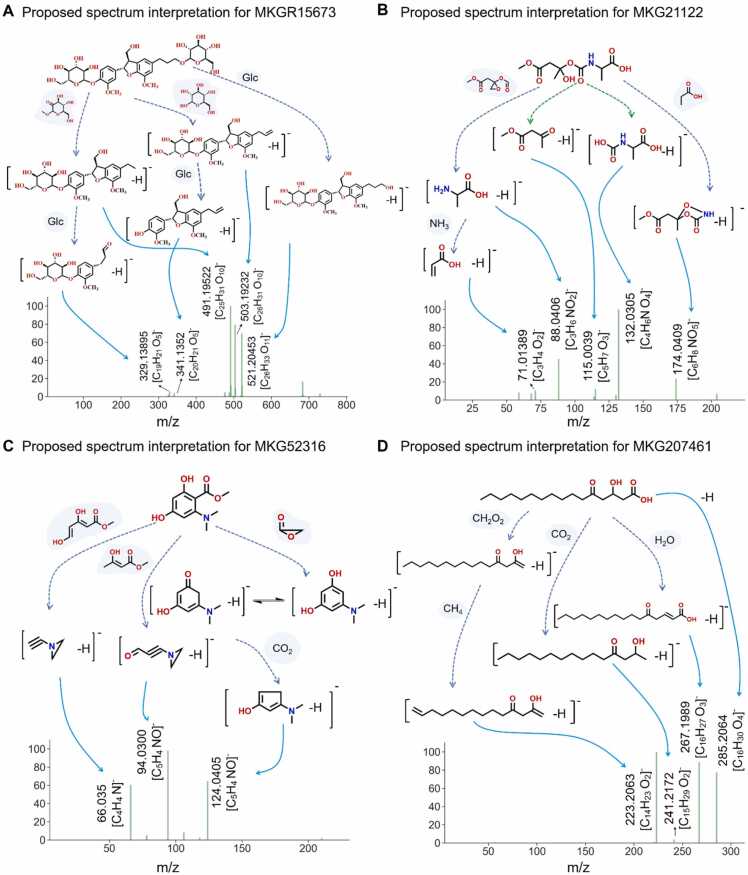
**Interpretation of potential novel compounds discovered by MassKG from various sources**. (A) Fragment trees and MS^2^ spectra of MKGR15673 from GB. (B) Fragment trees and MS^2^ spectra of MKG21122 from CP. (C) Fragment trees and MS^2^ spectra of MKG52316 from CP. (D) Fragment trees and MS^2^ spectra of MKG207461 from AM. The deep blue dashed line represents the fragment tree of these compounds, illustrating how they cleavage into smaller fragments. When two fragments are generated by the cleavage of the same bond, they are connected by a blue dashed line, indicating their shared origin. The light blue lines connect these fragments to their corresponding MS/MS spectra, facilitating the identification and verification of the compounds. Additionally, neutral losses, which occur during the fragmentation process, are marked with a grey shadow, providing further insights into the compound's structure and behavior during mass spectrometry analysis.

We further applied MassKG to analyze the metabolites of three herbal medicines, *Ginkgo biloba* (GB), *Codonopsis pilosula* (CP) and *Astragalus membranaceus* (Fisch) Bge. *var. mongholicus* (Bge.) Hsiao (AM) (analyzed in our pervious work [45])for systematically validation of MassKG in potential novel NP discovery. The MS^2^ data acquisition method for CP and AM was the same as described in Section 2.1, and both herbs were analyzed in negative ion mode. MS^2^ data of GB was reanalyzed from our previous work [46]. The annotation results of CP and AM were listed in Supplementary File 9–10 respectively. For each spectrum, the top 10 candidates were retrieved for 199 metabolites in CP and 170 metabolites in AM. As the fragment library was composed of both known and generated molecules, MassKG identified several potentially novel molecules. We would like to emphasize the new compound MKGR15673 identified the extract of GB leaves, which was isolated and analyzed by proton nuclear magnetic resonance (HNMR) in our group's previous work [46]. With reanalyzing by MassKG, we were surprised to find that the compound was successfully predicted and ranked first among candidates. This case fully demonstrates that using MassKG to analyze the NP chemical structures can explore a broader space, including the discovery of new structured NPs (Fig. 6A).

Additionally, two molecules from CP (MKG207461 and MKG52316, shown in Fig. 6B and Fig. 6C, respectively) and one molecule from AM (MKG207461, shown in Fig. 6D) were further presented to illustrate the novel NP structure discovery ability of MassKG. The major spectral features of these compounds were explained by the predicted fragments. Compound MKG207461 was matched to a deprotonated molecule peak of *m/z* 285.2064 [M-H]^-^ and the molecular formula was conducted as C_16_H_30_O_4_. The fragment ion peak was matched to *m/z* 223.2063, which was formed by neutral loss of CH_2_O_2_ and CH_4_ in the precursor structure; *m/z* 241.2172 was formed by the loss of CO_2_ in the precursor structure; *m/z* 267.1989 was formed by neutral loss of H_2_O in the precursor structure. Compound MKG21122 was matched with the addition ion *m/z* 294.0837 [M + FA-H] ^-^, and its molecular formula was inferred to be C_9_H_15_NO_7_. The matched fragment ion was *m/z* 88.0406, which was obtained by neutral loss of C_6_H_8_O_5_ in the precursor structure. This fragment further loses one molecule of NH_3_, resulting in fragment *m/z* 71.0139; The cleavage of the tertiary carbon C—O bond in the precursor ion resulted in two fragments, *m/z* 115.0039 and *m/z* 132.0305, respectively, based on the charged state of the fragments and the rearrangement of H atoms; The neutral loss of C_3_H_6_O_2_ in the mother nucleus structure resulted in fragments *m/z* 174.0409. Compound MKG52316 matched the deprotonated molecule peak *m/z* 210.0767, and its molecular formula was inferred to be C_10_H_13_NO_4_. It matched the fragment peak *m/z* 66.035, which was obtained by neutral loss of C_6_H_8_O_4_ from the precursor ion; The fragment peak *m/z* 94.0300 was obtained by the loss of C_5_H_8_O_3_ in the precursor structure; The fragment ion *m/z* 124.0405 lost one molecule of C_2_H_2_O_2_ through the precursor structure, resulting in a fragment structure where the phenolic hydroxyl group resonated as a carbonyl group, and then lost one molecule of CO_2_.

### 3.7 Implementing MassKG as a web server

**Fig. 7:**
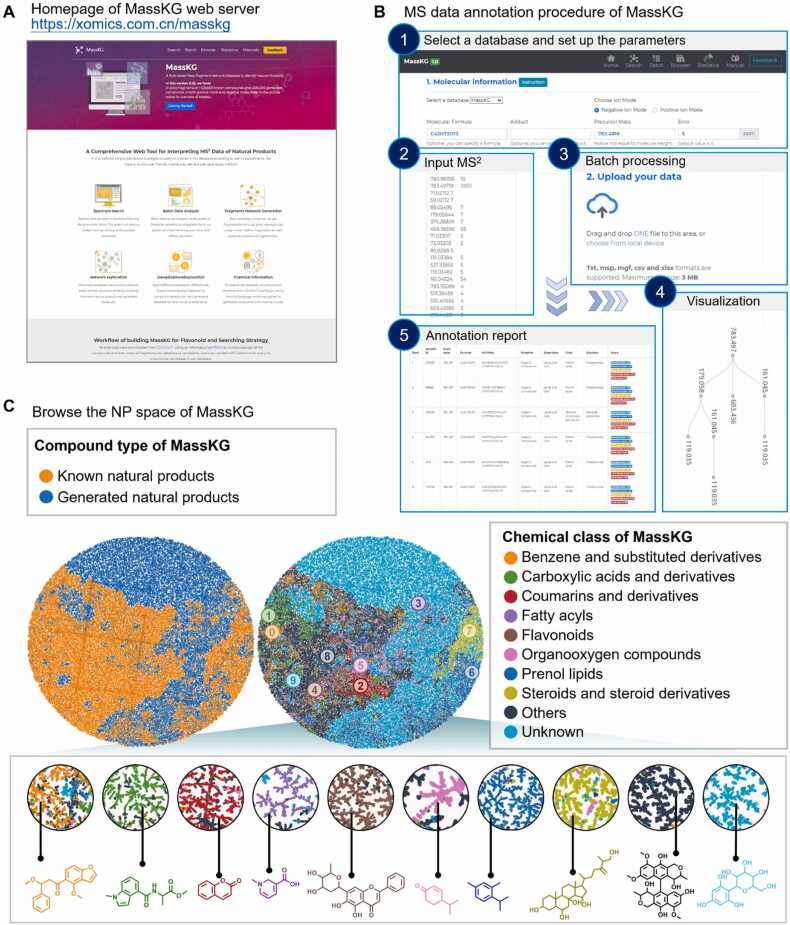
**Introduction to the MassKG web server.** (A) The homepage of MassKG with a user-friendly interface. (B) Demonstration of the single query tool and the batch query tools of MassKG. (C) Global visualization of the NP space of MassKG. Utilizing the TMAP approach [49], it displays over 400,000 known molecules and more than 250,000 generated molecules.

MassKG is designed to be an intelligent tool for the knowledge-based interpretation of metabolites based on MS2 data, with a particular focus on the fragmentation patterns of plant metabolites. We designed MassKG as a web server for a good availability of the aforementioned tools. In the current version, MassKG support both single spectra and batch MS file query modes as well as exploring the whole MassKG NP chemical space with a user-friendly interface (Fig. 7A). It can be used in conjunction with MS2 data processing software like MZmine [47] and MS-DIAL [48]. After raw data processing, the yielded feature table containing precursor *m/z* and MS^2^ spectra can be used to as the inputs of MassKG web server.

#### 3.7.1 Single query tool

The single search tool of MassKG web server was composed of four blocks. Frist, the candidate database was supposed to be selected. In this version, we designed three databases which named *MassKG* for the whole database, *coconut* for the known NPs and *generated* for only the novel predicted NPs respectively. The default database is *MassKG*. Then, the precursor mass and ion mode must be checked. The ppm block was set with a default value of 10. The molecular formula block and adduct block was not necessary since MassKG was able to calculate the candidate formula automatically. Next, users had to input the MS^2^ table in to the MS/MS text area. Once these procedures were finished, click the submit button and waiting for a second. The whole candidates will be gathered into a cleaned table, and their corresponding fragment trees would be constructed for a direct visualization (Fig. 7B).

#### 3.7.2 Batch query tool

In order to streamline the handling of batch inference tasks, we offer a comprehensive data reporting service. Users can upload MS^2^ data processed in formats like txt files generated by MS-DIAL software, msp files, or mgf files exported from MZmine. After uploading, users need to input three parameters: *TopK*, *threshold*, and *ppm*. Among these, *TopK* determines that the final report will retain the top K candidates for each MS^2^ spectra. *Threshold* is the peak area filtering threshold, and *ppm* is the mass error tolerance set for inferring molecular formulas from precursor *m/z* values. This module would return a metabolite annotation report (Fig. 7B).

#### 3.7.3 MassKG NP space browser

To facilitate users' exploration of the entire NP space of MassKG, we constructed a network graph of the NP space of MassKG utilizing TMAP [49]. The network was constructed based on structural similarity calculated by a string-based algorithm named MinHashed fingerprints (MHFP) [50]. The visualization of the TMAP was realized via Faerun [51]. The TMAP of MassKG were consisted of about 660,000 known and generated molecules and were displayed in 6 different labels, which were molecule type, chemical class, heavy atom counts, C atom fraction, ring atom fraction and largest ring size. The first label, molecule type, discriminant the molecules by the known NPs from COCONUT database and the generated NPs (Fig. 7C). It can be observed that the distribution of known generated NP structures overlaps partially, while another part is relatively separated.

The right panel shows the top 8 categories ranked by abundance of chemical class calculated by Classyfire [38], including *Benzene and substituted derivatives*, *carboxylic acid*s *and derivatives*, *coumarins and derivatives*, *fatty acyls*, *flavonoids*, *organooxygen compounds*, *prenol lipids and Steroids and steroid derivatives*. The other chemical classes were labeled other, while the generated molecules were labeled with unknown for the reason of ontology unavailability. The result showed that *coumarins and derivatives*, *flavonoids* and *steroids and steroid derivatives* were well clustered, while others were not. The TMAP labeled with heavy atom counts (HAC), C atom fraction, ring atom fraction and largest ring size were illustrated in Fig. S6–S9.

## 4 Discussion

LC-MS/MS is a key technology for identifying NP structures from complex systems [52], [53]. However, whether for dereplicating known NPs or discovering novel structures, analyzing MS^2^ data remains challenging. MassKG addresses this challenge by employing a knowledge-based in silico fragmentation model and a molecule generation model, streamlining the intricate process of MS^2^ data annotation. The hallmark of knowledge-based approaches is their reproducibility and interpretability, offering distinct advantages over data-driven methods that often grapple with adaptability and scalability to new datasets [25]. MassKG exhibits consistent and robust performance in predicting a wide array of NP structures, including novel ones, without necessitating retraining. MassKG further provides clear and interpretable annotation results, shedding light on the fragmentation patterns within complex NP MS^2^ data. This not only deepens scientists' comprehension of the characteristics of NP MS^2^ data but also significantly enhances the precision of structure identification [14], [54]. These innovations pave the way for intelligent discovery and prediction of fragmentation patterns, substantially elevating the efficiency and accuracy of metabolite annotation processes.

However, given the limited availability of open-source data from various vendors, equipment, and analysis conditions, the summarized fragmentation knowledge may exhibit biases. As such, understanding fragmentation patterns is an ongoing process requiring continuous updates and optimizations. As our analytical work progresses and data accumulates, we will continually refine and enhance our models to improve their accuracy and applicability. Additionally, the fragment generator and candidate scoring function will be optimized as the test dataset expands.

Additionally, there are differences between the dereplication task and the novel discovery task. Research has demonstrated that incorporating a score related to candidate references can enhance annotation accuracy for known compounds [42], [55]. However, adding a meta score may hinder the discovery of novel candidates that share the same molecular formula as well-known natural products, as their meta score would be 0. Therefore, we recommend that users apply a meta score for accurate and rapid dereplication, while disregarding it for suspect spectra of potential novel structures.

To the best of our knowledge, this represents the first study to apply generation models to enrich the chemical space of NPs. While MassKG have successfully generated over 200,000 novel structures, including experimentally verified cases in GB, we have to recognize that we cannot conclusively assert that all generated structures represent viable NPs. MassKG has offered plausible explanations for fragmentation patterns in potential new structures discovered in CP and AM, though further isolation, preparation, and experimental studies, such as HNMR, are necessary for confirmation. Nonetheless, with the ascendancy of large language models, we believe that these generation models will play an increasingly crucial role in metabolomics and the discovery of novel NP structures in the future.

The MassKG web server currently focuses on MS^2^ data annotation but plans to incorporate an online fragmentation module in the future. The MassKG web server will be updated weekly to ensure its currency and relevance.

## 5 Conclusion

The core target for LC-MS/MS in NP study is to discovery valuable NP efficiently, which requires quick dereplication and effort on novel NP investigation. MassKG is composed of a knowledge-based NP fragment generator and an RNN-based molecule generator for annotating the MS^2^ data of NPs intelligently. The candidate database of MassKG comprises 407,720 known NPs from 43 different classes, as well as 266,353 novel compounds generated by a molecule generation model. The in silico fragments of these compounds were generated to construct the MassKG spectra library, which is further used for MS^2^ data annotating coupled with a candidate ranking function. By a baseline study, MassKG showed outperformed annotation accuracy than the state-of-the-art algorithms. In practical applications, MassKG was applied to annotate the metabolites of GB, CP and AM and successfully predicted a novel structure from GB that was absence from the current database. The MS^2^ spectra of other three potential novel NPs from CP and AM were also well annotated. MassKG has been implemented as a web server to facilitate MS^2^ data annotation process. Overall, this study contributes valuable insights and tools to the field of MS based metabolomics and NP discovery.

## CRediT authorship contribution statement

**Yi Zhong:** Validation, Methodology. **Zhenhao Li:** Writing – original draft, Validation, Formal analysis, Data curation. **Huihui Liu:** Validation. **Zehua Jin:** Validation, Formal analysis. **Xiaohui Fan:** Writing – review & editing, Supervision, Conceptualization. **Jie Liao:** Writing – review & editing, Supervision, Conceptualization. **Bingjie Zhu:** Writing – original draft, Visualization, Methodology, Formal analysis. **Yugang Lin:** Validation. **Shufang Wang:** Validation. **Haoran Li:** Software. **Tianhao Wang:** Software. **Tianhang Lv:** Software. **Zhiwei Ge:** Resources. **Tianyi Ma:** Software.

## Declaration of Competing Interest

None.

## Data Availability

The in-house experimental datasets for NPs and natural products as well as the generated 266,353 novel structures are available at https://github.com/SpaTrek/MassKG. The COCONUT dataset is open source and can be freely download at https://coconut.naturalproducts.net/download. Curated MassKG database containing known and generated compounds is available at https://drive.google.com/file/d/1gJdIZ63MXCYHwd6eMDUJopDXPIvZRYSB/view?usp=drive_link.
