# Supplementary material for: Knowledge-based in silico fragmentation and annotation of mass spectra for natural products with MassKG
Source: Comput Struct Biotechnol J. 2024 Sep 7;23:3327–41. doi: 10.1016/j.csbj.2024.09.001 (PMC11415640; doi:10.1016/j.csbj.2024.09.001)
Supplement: Supplementary file 12 — Supplementary material [file mmc12.docx]

Supplementary file for “Knowledge-based In Silico Fragmentation and Annotation of Mass Spectra for Natural Products with MassKG”

# Experimental method of in-house data acquisition

## Sample preparation for LC-MS analysis

The stock solutions of 257 standard compounds were prepared by dissolve them in 70% methanol at concentrations of 0.1-1mg/mL. Then these stock solutions were mixed up to a proper final concentration for LC-MS analysis.

The herbal medicine samples were thoroughly ground and extracted using ultrasonic extraction with methanol for 1 hour. After centrifugation, the supernatant was collected for analysis.

## LC-MS analysis

Chromatography separation was achieved with Waters ACQUITY UPLC HSS T3 column (1.8 μm particle size, 150 mm × 2.1 mm). Data acquisition was performed on an AcquityTM UPLC system (Waters Technologies, U.S.A) coupled to an UPLC-Triple-TOF/MS system (6600+ QTof, SCIEX, Waters technologies, U.S.A). The mobile phase A was water with 0.1% formic acid and mobile phase B was acetonitrile with 0.1% formic acid. Gradient elution conditions: 0.00-25.00 min, 5-40 % B; 25.00-35.00 min,40-95 % B; 35.00-37.00 min,95 % B, The flow rate was 0.300 mL/min, the column temperature was 50℃, and the injection volume was 3μL. Accurate mass measurement was carried on both negative ion mode and positive ion mode in conditions as follows: scan range: m/z 100-1500; gas 1 pressure (GS1): 55 psi; gas 2 pressure (GS2): 55 psi; curtain gas (CUR): 35 psi; ion source temperature (TEM): 600℃ (positive) and 550℃ (negative); ion source voltage (IS): 5500 V (positive) - 4500 V (negative); first scan: declustering potential (DP): 100 V; collision energy (CE): 10V; second scan: TOF MS-Product Ion-IDA mode for mass spectrometry data acquisition, with CID energy of 40 ±20 eV. Before injection, a CDS pump is used for mass axis calibration to ensure a mass axis error of less than 2 ppm.

# Molecular network of in-house dataset in the positive ion mode


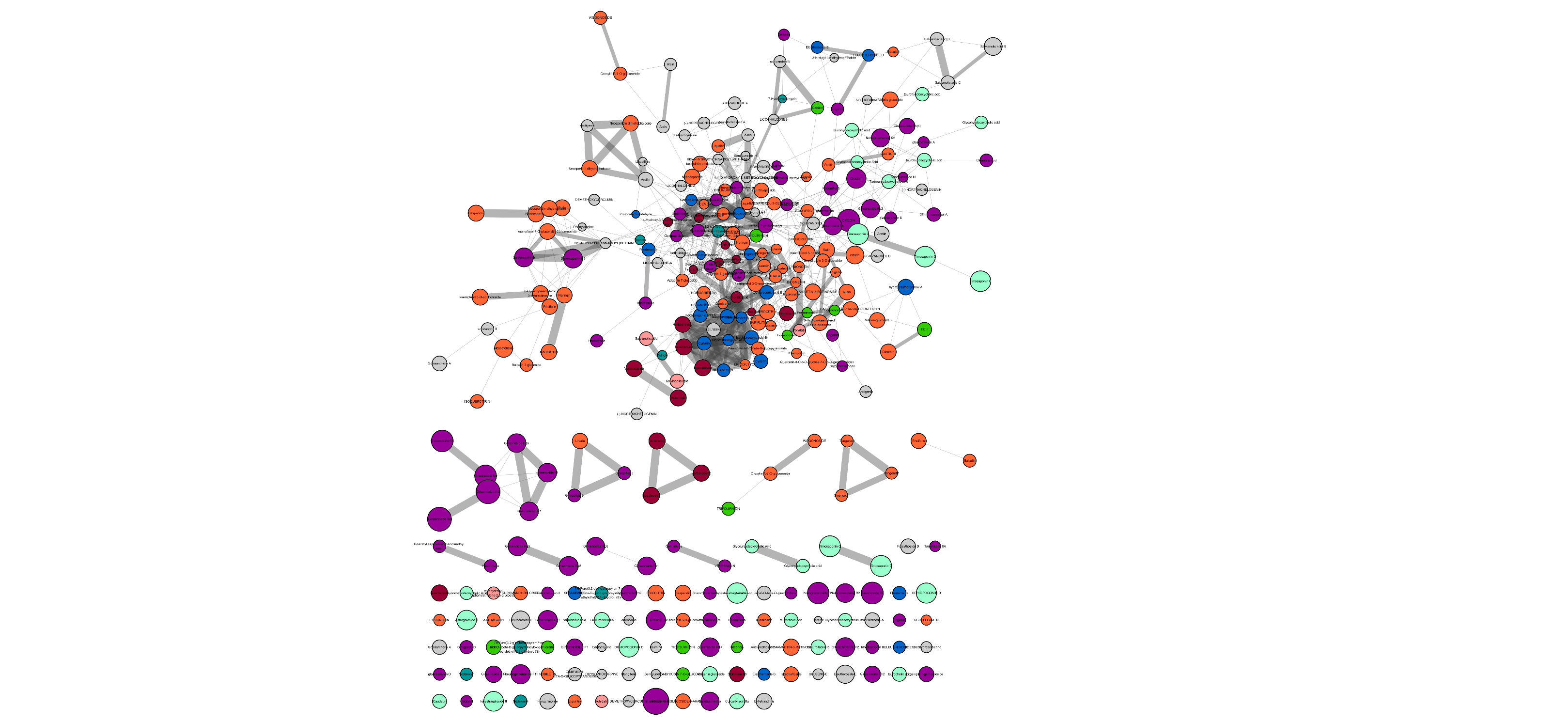


Fig S1. The molecule network of in-house standards in positive ion mode.

# Fragmentation

The correlations between the precursor m/z, the number of fragments, and the number of simulated MS^2^ peaks of NPs processed by the trained fragment generator were 0.905, 0.922, and 0.992 in positive ion mode, and 0.711, 0.71, 0.993 in negative ion mode (Fig. S2A). It can be observed that in both positive and negative ion modes, the number of fragments/peaks positively correlates with the m/z (m/z) of the precursor ion, with a higher correlation in positive ion mode (>0.9). This aligns with the fundamental principle of our fragment generator, which is that larger molecules theoretically have more chemical bonds that can be broken, resulting in more fragment ions. However, in our experimental data, the presence of [2M-H]^-^ ion types in negative ion mode leads to higher m/z values and fewer fragments generated from virtual cleavage of the actual molecular structure, thus reducing the correlation. There is a significant correlation between the number of fragments and the number of peaks in the simulated spectrum, with the number of peaks being approximately 2-3 times the number of fragments. This is a result of our design for H rearrangement and charge distribution, where the same neutral fragment structure may have different H rearrangements, resulting in multiple m/z values. Specifically, for the tested NPs, the outlier in positive ion mode is Kuromanin chloride (Fig. S2B). Experimentally, it was observed to have an m/z of 485.0813 with [M+H]^+^ ion type. However, its SMILES structure contains both positive and negative charges, which MassKG currently not follow with interest.


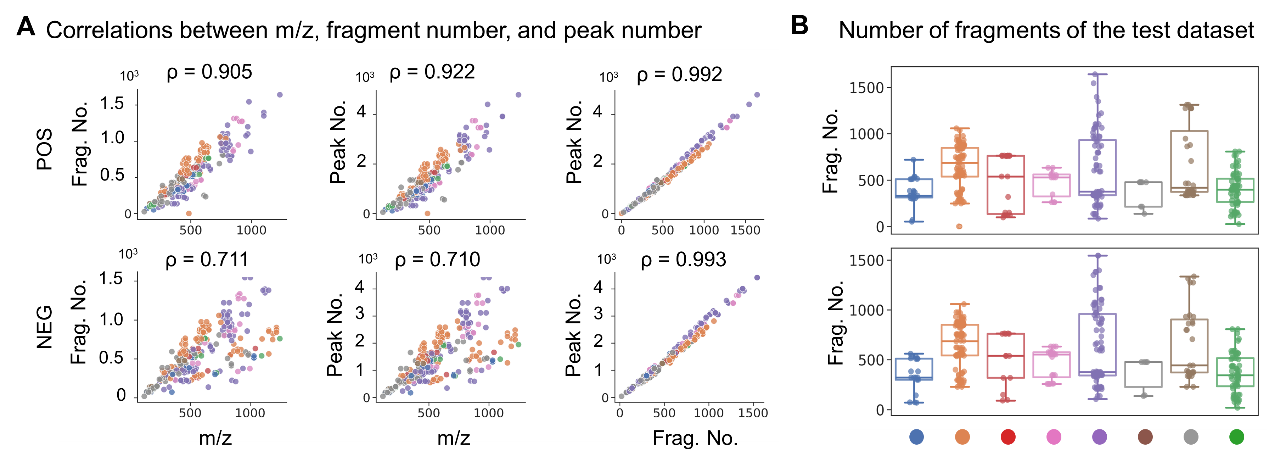


**Fig S2 Fragment generation performance of the MassKG.** (A) Assessment of the capability of MassKG in predicting fragments and MS^2^ peaks, showcase the correlations between m/z values, molecular fragment numbers, and mass spectrometry peak numbers. (B) The number of fragments generated for each chemical class in test dataset.

# Details for ranking score design

## BDE prediction model

The random forest model was trained by the training dataset of GNPS library. The model input is built through a series of steps that involve extracting features from molecular structures, specifically focusing on bonds and atoms. Here’s a summary of the features used for bond and atom embeddings:

**Atom Features**

1. Atom Symbol

- One-hot encoding for the atom type (e.g., C, H, O, N, P, S, Un), where Un represents the unknown symbols.

2. Implicit Valence

- One-hot encoding for the implicit valence of the atom (0 to 4).

3. Degree

- One-hot encoding for the degree of the atom (0 to 6).

4. Formal Charge

- One-hot encoding for the formal charge of the atom (-1, 0, 1).

5. Hybridization

- One-hot encoding for the hybridization state of the atom (SP, SP2, SP3, SP3D).

**Bond Features**

1. Bond Type

- Boolean features indicating the type of bond (single, double, triple, aromatic).

2. Conjugation

- A boolean feature indicating if the bond is conjugated.

3. Ring Membership

- A boolean feature indicating if the bond is part of a ring.

## Parameter optimization

The optimization target is the top 1 annotation accuracy on the in-house dataset, for both positive and negative ion mode. Each parameter ranges from 0.1 to 1. Then the parameters with highest top 1 accuracy in both positive and negative ion mode was selected for the final score function.


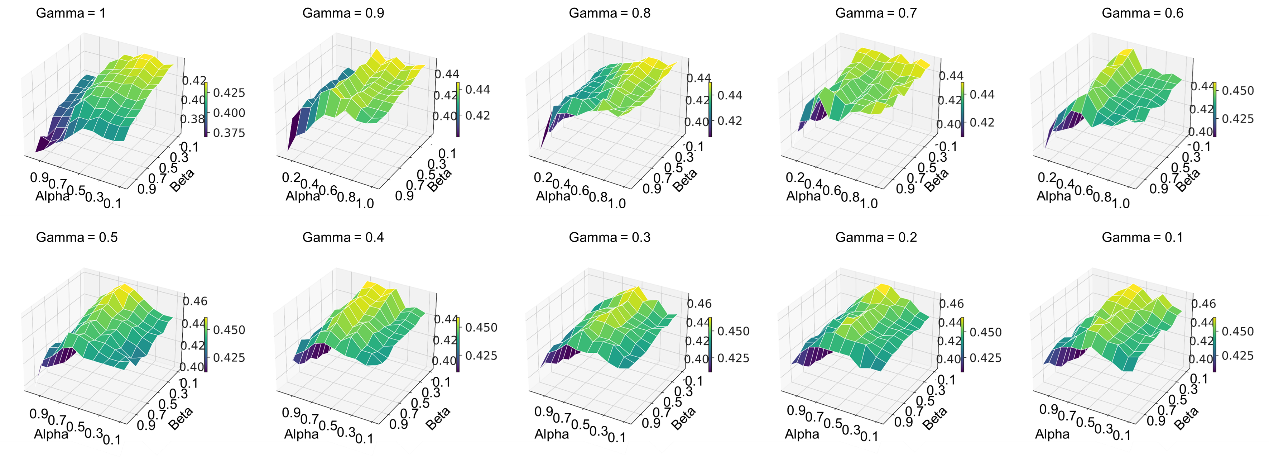


**Fig S3. Grid search optimization of parameter alpha, beta and gamma.**

## Predicted spectra

We showcase the predicted spectra with highest similarities to the experimental spectra in Fig S4 and Fig S5.


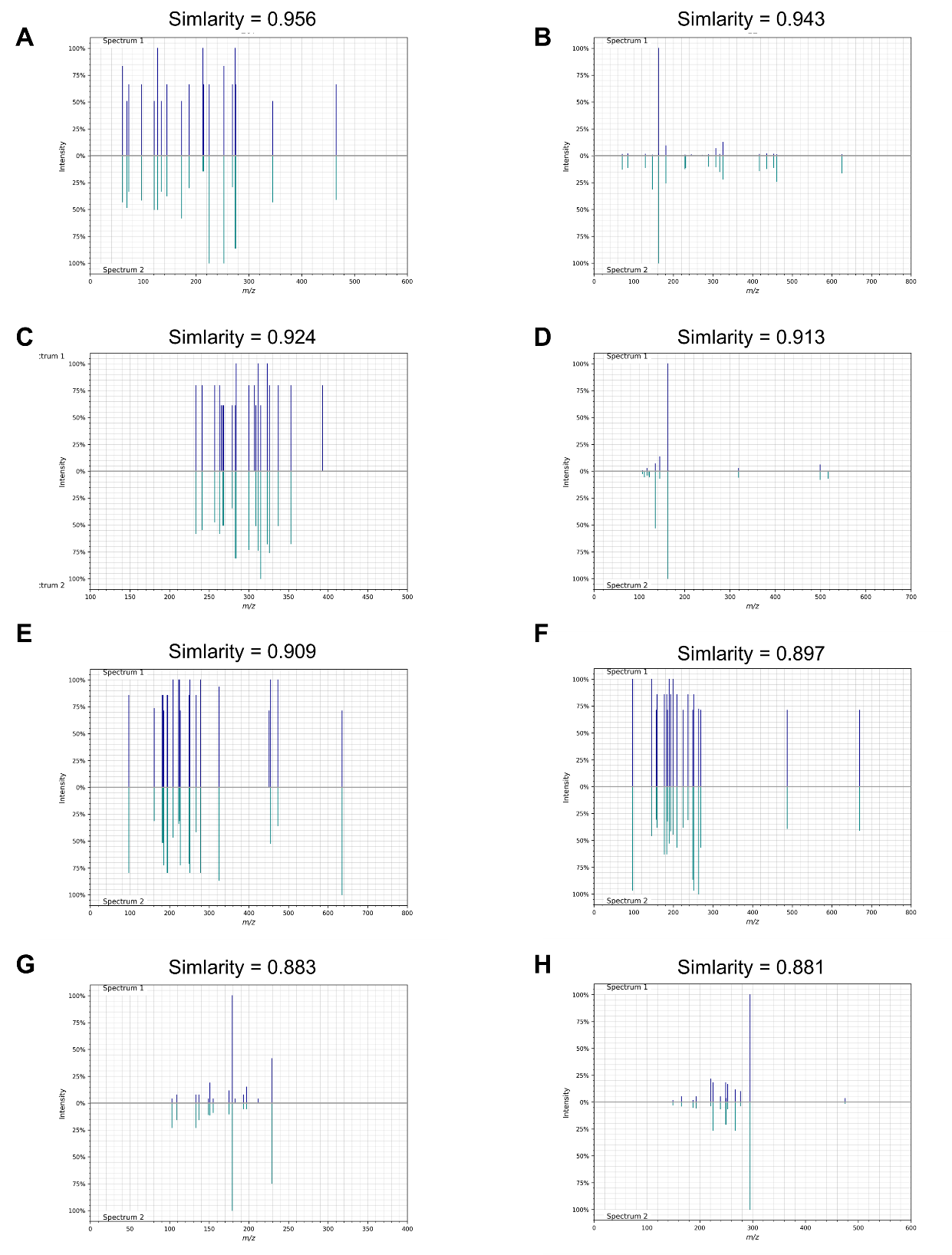


**Fig S4. Examples of predicted MS^2^ spectra of MassKG compared against experimental spectra in positive ion mode, titled with cosine similarity.**


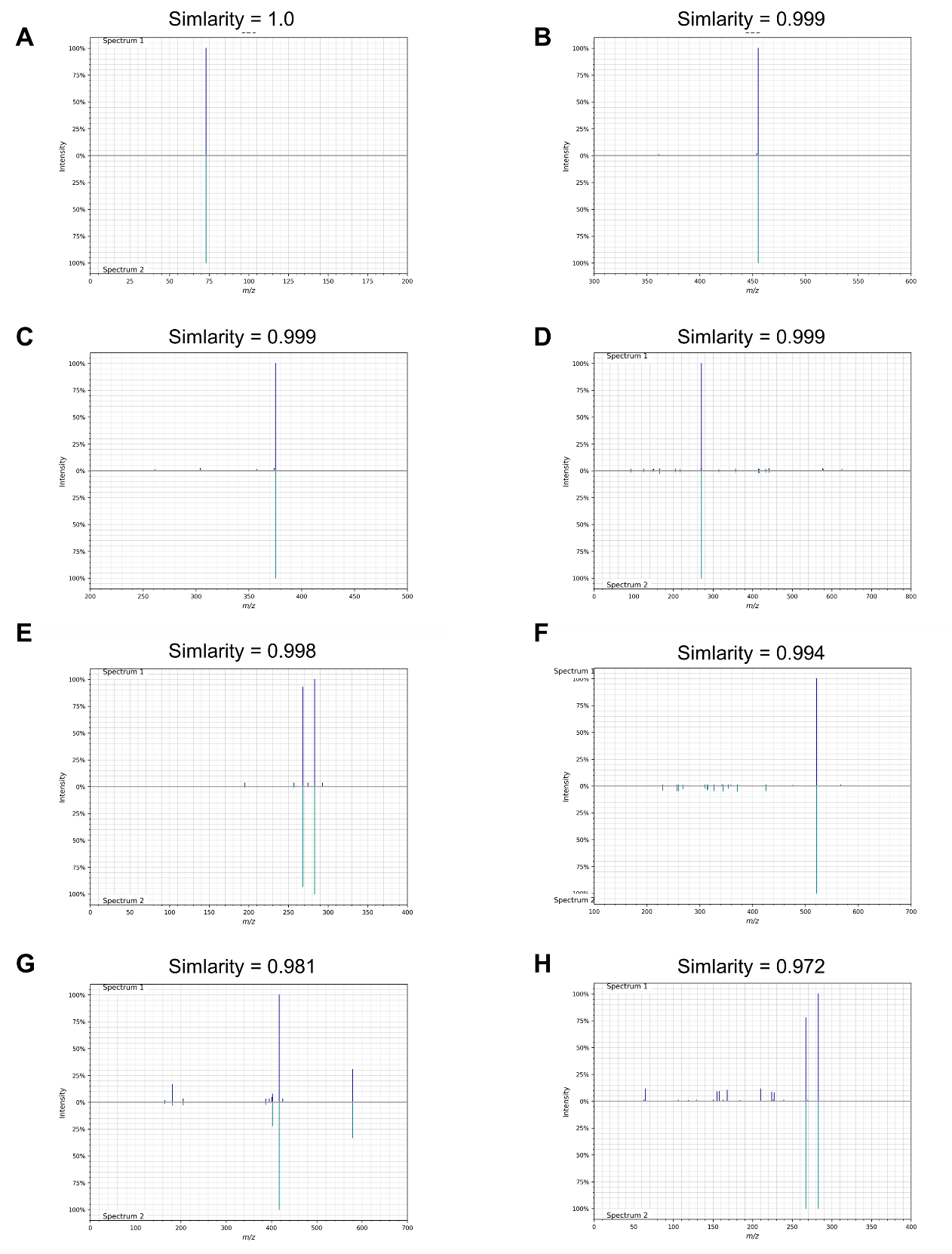


**Fig S5. Examples of predicted MS^2^ spectra of MassKG compared against experimental spectra in negative ion mode, titled with cosine similarity.**

# Metrics of the molecule properties for molecular generator

The basic molecular characteristics were calculated by the rdkit.Chem.Descriptors module, including the molecular weight, the topological polar surface area (TPSA), QED, LogP.

The logarithm of the partition coefficient (LogP), is a measure of a compound's lipophilicity or hydrophobicity. It represents the ratio of a compound's concentration in a nonpolar solvent (usually octanol) to its concentration in a polar solvent (usually water) at equilibrium. LogP is widely used in medicinal chemistry and drug design to predict a molecule's behavior in biological systems, including its absorption, distribution, metabolism, and excretion (ADME) properties.

QED (Quantitative Estimation of Drug-likeness) is a molecular descriptor used in cheminformatics to assess the drug-likeness of a molecule[1]. It provides a quantitative measure indicating how "drug-like" a molecule is based on various molecular properties associated with favorable pharmacokinetic and pharmaceutical characteristics. The empirical rationale of the QED measure reflects the underlying distribution of molecular properties including molecular weight, logP, topological polar surface area, number of hydrogen bond donors and acceptors, the number of aromatic rings and rotatable bonds, and the presence of unwanted chemical functionalities.

percentage of sp3 carbons, percentage of heteroatoms, number of rings were calculated by the rdkit.Chem.Lipinski module.

BertzTC was calculated by the rdkit.Chem.GraphDescriptors module. A topological index meant to quantify “complexity” of molecules.Consists of a sum of two terms, one representing the complexity of the bonding, the other representing the complexity of the distribution of heteroatoms[2].

Natural product-likeness score was calculated by the npscore module of rdkit.

Table S1 Metrics for evaluation the molecular generation model.

| **Outcome** | **Value** |
| --- | --- |
| % valid | 0.510424 |
| % novel | 0.927192 |
| % unique | 0.909034 |
| KL divergence, atoms | 0.002994 |
| Jensen-Shannon distance, atoms | 0.026476 |
| Wasserstein distance, atoms | 0.0042 |
| KL divergence, MWs | 0.415221 |
| Jensen-Shannon distance, MWs | 0.31325 |
| Wasserstein distance, MWs | 9.04E-05 |
| KL divergence, logP | 0.065453 |
| Jensen-Shannon distance, logP | 0.130757 |
| Wasserstein distance, logP | 0.001938 |
| KL divergence, Bertz TC | 0.488133 |
| Jensen-Shannon distance, Bertz TC | 0.3414 |
| Wasserstein distance, Bertz TC | 3.62E-05 |
| KL divergence, QED | 0.096088 |
| Jensen-Shannon distance, QED | 0.163105 |
| Wasserstein distance, QED | 0.287899 |
| KL divergence, TPSA | 0.212536 |
| Jensen-Shannon distance, TPSA | 0.232932 |
| Wasserstein distance, TPSA | 0.000165 |
| Internal diversity | 0.084678 |
| External diversity | 0.086749 |
| KL divergence, # of rings | 0.385512 |
| KL divergence, # of aliphatic rings | 0.072421 |
| KL divergence, # of aromatic rings | 0.107946 |
| Jensen-Shannon distance, # of rings | 0.26478 |
| Jensen-Shannon distance, # of aliphatic rings | 0.115685 |
| Jensen-Shannon distance, # of aromatic rings | 0.16251 |
| Wasserstein distance, # of rings | 0.051923 |
| Wasserstein distance, # of aliphatic rings | 0.051923 |
| Wasserstein distance, # of aromatic rings | 0.035284 |
| KL divergence, SA score | 0.149238 |
| Jensen-Shannon distance, SA score | 0.201943 |
| Wasserstein distance, SA score | 0.026241 |
| KL divergence, NP score | 0.127829 |
| Jensen-Shannon distance, NP score | 0.184643 |
| Wasserstein distance, NP score | 0.035223 |
| KL divergence, % sp3 carbons | 0.00842 |
| Jensen-Shannon distance, % sp3 carbons | 0.045143 |
| Wasserstein distance, % sp3 carbons | 0.057126 |
| KL divergence, % rotatable bonds | 0.077083 |
| Jensen-Shannon distance, % rotatable bonds | 0.133667 |
| Wasserstein distance, % rotatable bonds | 0.294362 |
| KL divergence, % stereocenters | 0.049061 |
| Jensen-Shannon distance, % stereocenters | 0.112078 |
| Wasserstein distance, % stereocenters | 0.307173 |
| KL divergence, Murcko scaffolds | 5.557671 |
| Jensen-Shannon distance, Murcko scaffolds | 0.680345 |
| Wasserstein distance, Murcko scaffolds | 2.86E-06 |
| KL divergence, hydrogen donors | 0.041352 |
| KL divergence, hydrogen acceptors | 0.033382 |
| Jensen-Shannon distance, hydrogen donors | 0.097792 |
| Jensen-Shannon distance, hydrogen acceptors | 0.089541 |
| Wasserstein distance, hydrogen donors | 0.016385 |
| Wasserstein distance, hydrogen acceptors | 0.008548 |

# TMAP of the chemical space

The heavy atom counts (HAC), C atom fraction, ring atom fraction, and largest ring size of each molecule in the MassKG are calculated as a supplementary description of the chemical space. The statistic results of these molecular properties are listed in Table S2. The HAC within the expansive MassKG chemical space exhibits a wide range, spanning from a minimum of 4 atoms to a maximum of 209 atoms per molecule. This significant variation underscores the diversity of molecular structures captured within the dataset. Notably, the majority of molecules in the space possess a HAC value less than 34, indicating that the distribution is skewed towards smaller and moderately-sized molecules. For the fraction of carbon atoms (C frac), molecules consist of approximately 50% carbon atoms on average. The smallest fraction of carbon atoms in any molecule is 0%, indicating at least one molecule contains no carbon. There is a interesting observation that the median value of C frac is exactly 0.5, which also happens to be one of the quartiles (Q1 and Q3 being 0.25 and 0.75, respectively). The ring atom fraction (RAF) has a Q1 of 0.45, a median of 59%,and a Q3 of 0.70. For the largest ring size (LRS), it is obviously to find that the majority of molecules have the largest ring size of exactly 6 atoms, since the Q1, median and Q3 are all equal to 6.

Table S2 Statistic of the molecular properties.

|  | **HAC** | **C frac** | **RAF** | **Largest ring size** |
| --- | --- | --- | --- | --- |
| mean | 29.87 | 0.50 | 0.55 | 6.29 |
| std | 16.51 | 0.29 | 0.22 | 3.24 |
| min | 4 | 0.00 | 0.00 | 0 |
| Q1 | 20 | 0.25 | 0.45 | 6 |
| median | 26 | 0.50 | 0.59 | 6 |
| Q3 | 34 | 0.75 | 0.70 | 6 |
| max | 209 | 0.99 | 1.00 | 78 |


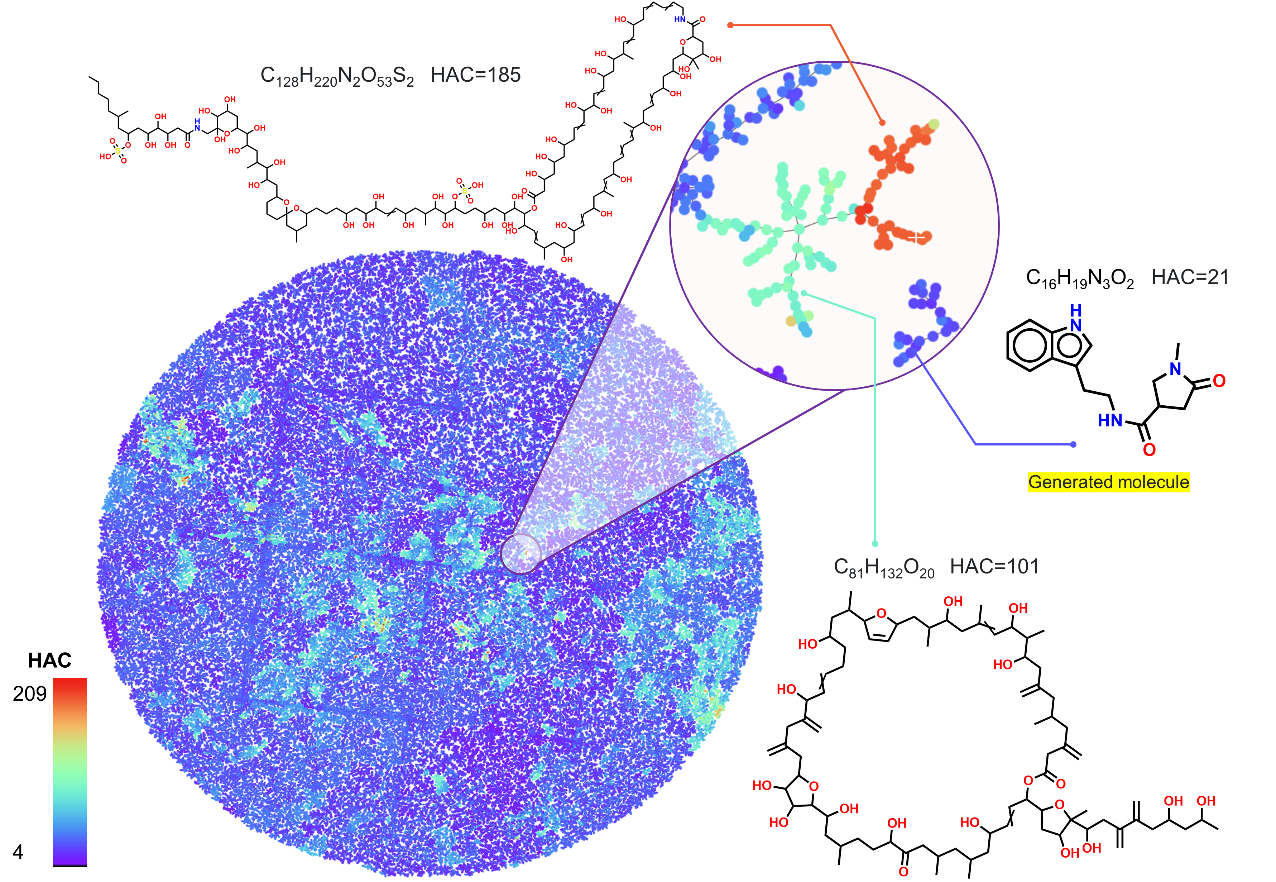


Fig. S6. TMAP of the HAC of the whole MassKG chemical space. HAC value of the molecules range for 4 to 209 and most molecules have a HAC value less than 34. A sub cluster with a molecule has HAC 185, a molecule has HAC 101 and a generated molecule with HAC 21 are showcased.


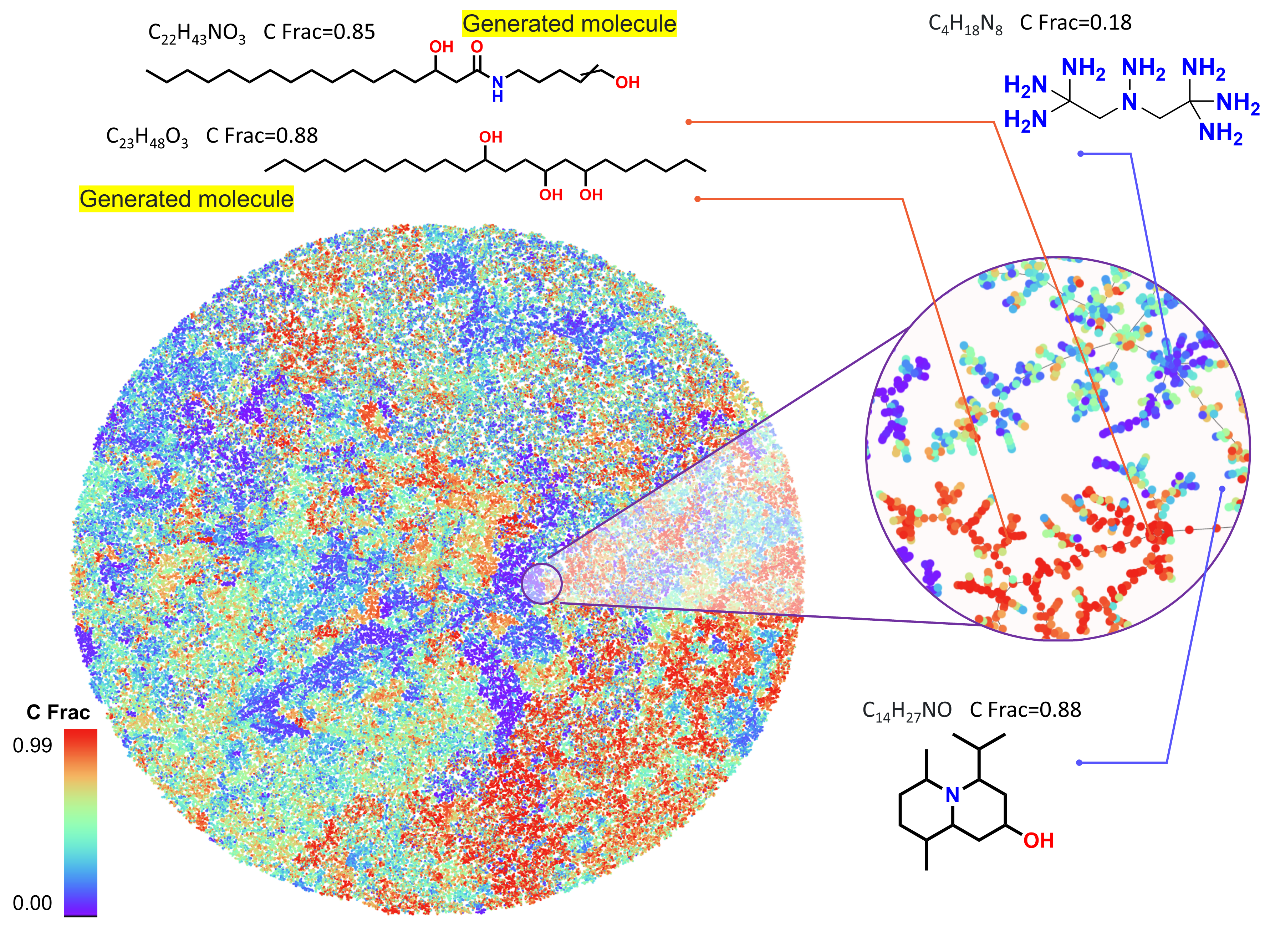


Fig. S7 TMAP of the C frac of the whole MassKG chemical space. C Frac value of the molecules range for 0.00 to 0.99. Overall, the distribution of C Frac is relatively uniform. A sub cluster with two generated molecules with C Frac of 0.85 and 0.88, as well as two known molecules with C Frac of 0.18 and 0.88 are showcased.


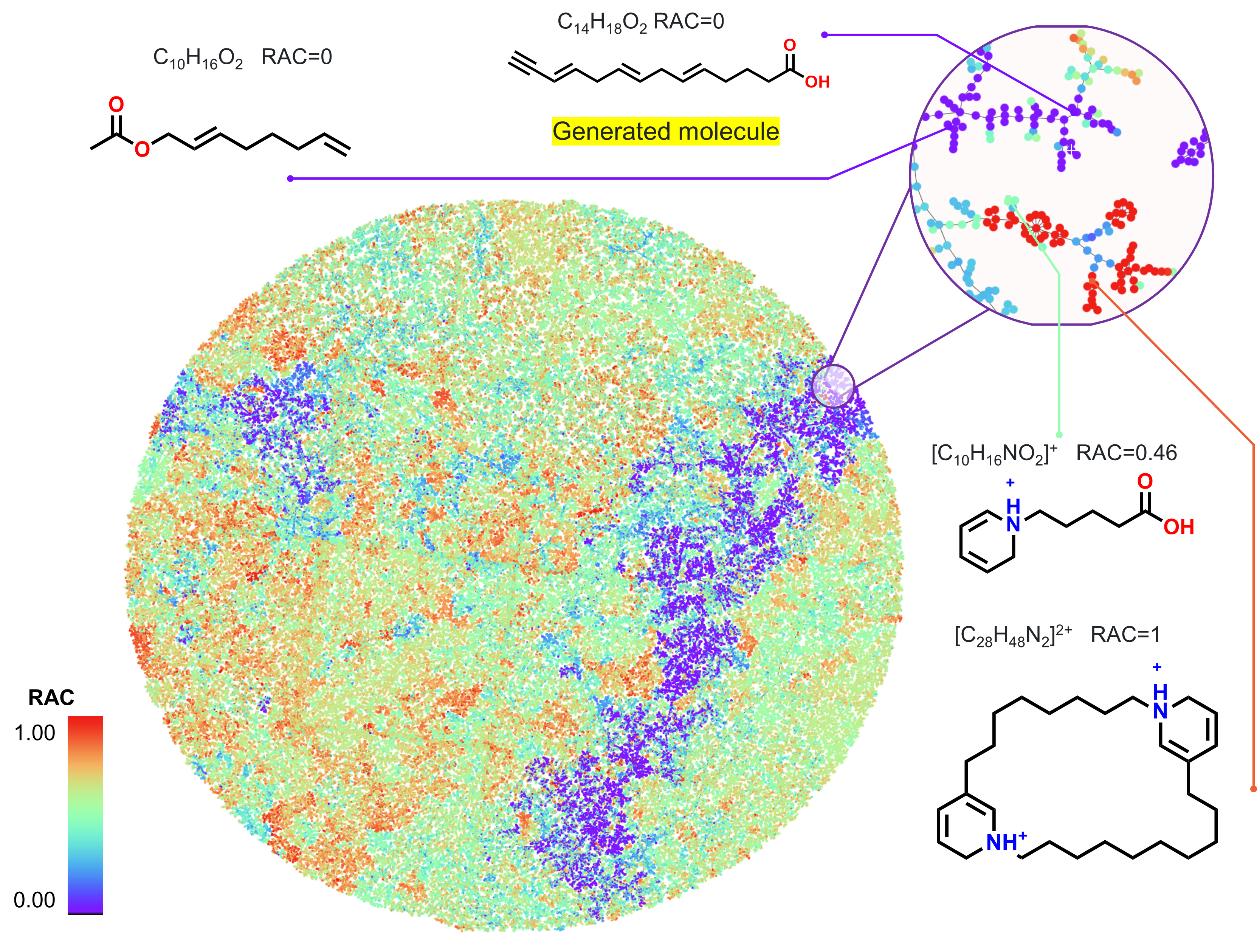


Fig. S8 TMAP of the RAF of the whole MassKG chemical space. RAF ranges from 0.00 to 1.00. A known molecule and a generated molecule with RAC of 0 are exhibited, which means they have a chain structure. Molecule with RAC of 0.46 and 1 are also selected as examples.


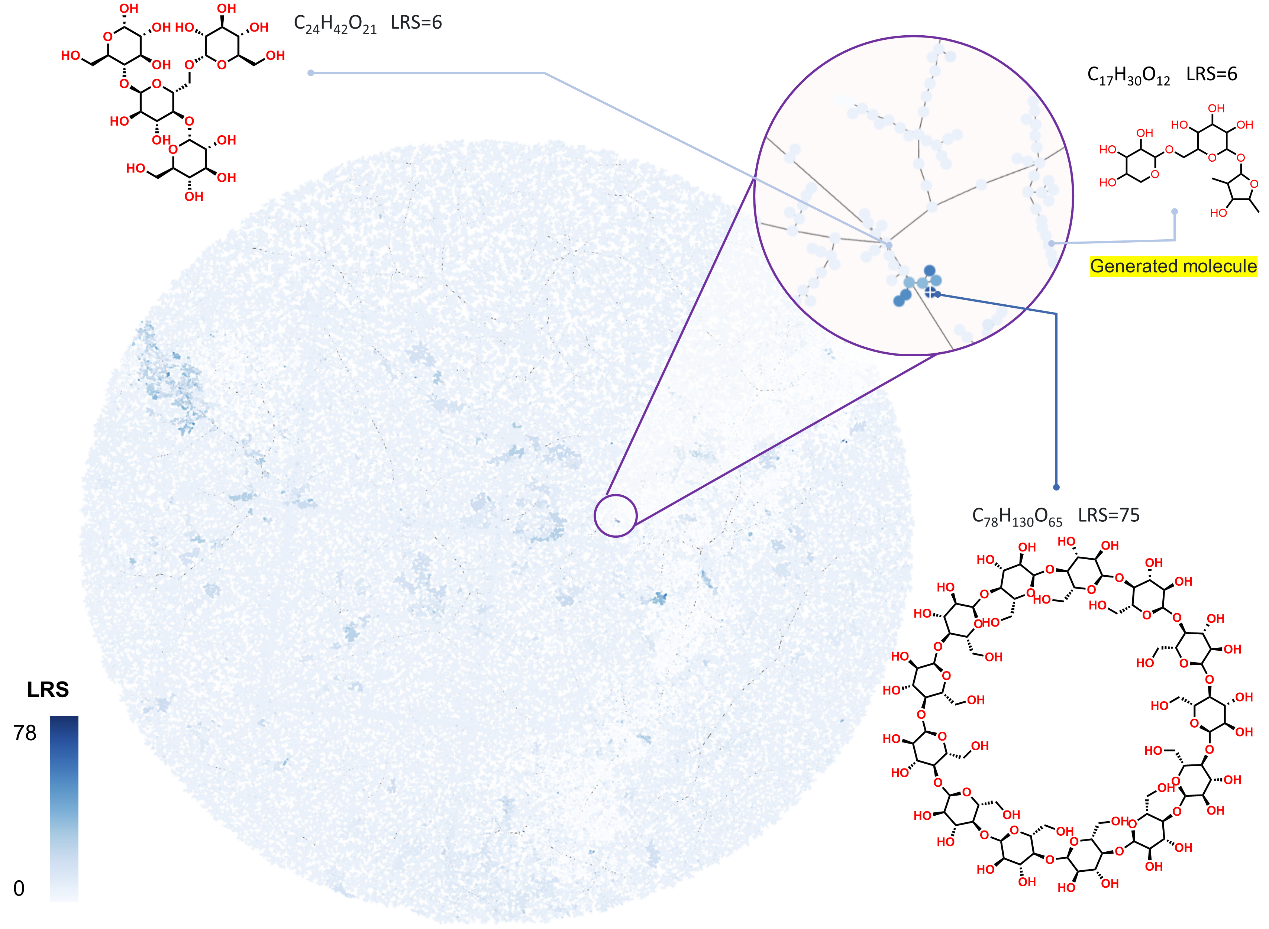


Fig. S9 TMAP of the LRS of the whole chemical space. The LRS ranges from 0 to 78 while most molecules have LRS of 6. Here a known molecule and a generated molecule with LRS of 6 are showcased, as well as a molecule with large LRS of 75. The generated molecules seldom has a larger LRS than 6.

**References:**

[1]. Bickerton, G.R., et al., Quantifying the chemical beauty of drugs. Nature Chemistry, 2012. 4(2): p. 90-98.

[2]. Bertz, S.H., On the complexity of graphs and molecules. Bulletin of Mathematical Biology. 45(5): p. 849-855.
